# Supplementary material for: Immune signature of Chlamydia vaccine CTH522/CAF®01 translates from mouse-to-human and induces durable protection in mice
Source: Nat Commun. 2024 Feb 23;15:1665. doi: 10.1038/s41467-024-45526-2 (PMC10891140; doi:10.1038/s41467-024-45526-2)
Supplement: Supplementary file 1 — Supplementary Information [file 41467_2024_45526_MOESM1_ESM.pdf]

Supplementary information

CTH522	CT681 Peptide nr.	Overlapping peptide sequences covering CTH522[a]	recognized by % donors[b]	% aa conserved across serovar[c]
CT681^{D}34-259: CTH523	CT681 P4 D	TWCDAISMRVGYYGDFVFDR	40	
	CT681 P5 D	GYYGDFVFDRVLKTDVNKEF	30	
	CT681 P6 D	VLKTDVNKEFQMGAKPTTDT	20	
	CT681 P7 D	QMGAKPTTDTGNSAAPSTLT	10	
	CT681 P8 D	GNSAAPSTLTARENPAYGRH	10	
	CT681 P9 D	ARENPAYGRHMQDAEMFTNA	10	
	**CT681 P10 D**	**MQDAEMFTNAACMALNIWDR**	**50**	**95-100**
	CT681 P11 D	ACMALNIWDRFDVFCTLGAT	40	
	CT681 P12 D	FDVFCTLGATSGYLKGNSAS	0	
	CT681 P13 D	SGYLKGNSASFNLVGLFGDN	30	
	CT681 P14 D	FNLVGLFGDNENQKTVKAES	0	
	CT681 P15 D	ENQKTVKAESVPNMSFDQSV	30	
	CT681 P16 D	VPNMSFDQSVVELYTDTTFA	30	
	CT681 P17 D	VELYTDTTFAWSVGARAALW	30	
	CT681 P18 D	WSVGARAALWECGCATLGAS	10	
	**CT681 P19 D**	**ECGCATLGASFQYAQSKPKV**	**60**	**95-100**
	**CT681 P20 D**	**FQYAQSKPKVEELNVLCNAA**	**60**	**95-100**
	CT681 P21 D	EELNVLCNAAEFTINKPKGY	10	
	CT681 P22 D	EFTINKPKGYVGKEFPLDLT	20	
	CT681 P23 D	VGKEFPLDLTAGTDAATGTK	30	
	CT681 P24 D	AGTDAATGTKDASIDYHEWQ	20	
	CT681 P25 D	DASIDYHEWQASLALSYRLN	30	
extVD4^{D}extVD4^{E}extVD4^{F}extVD4^{G}:CTH518	CT681 P26 DE	ASLALSYRLNMFTPYIGVKW	10	
	CT681 P27 DE	MFTPYIGVKWSRASFDADTI	10	
	CT681 P28 DE	SRASFDADTIRIAQPKSATA	20	
	CT681 P29 DE	RIAQPKSATAIFDTTTLNPT	20	
	CT681 P30 D	IFDTTTLNPTIAGAGDVKTG	40	
	CT681 P31 D	IAGAGDVKTGAEGQLGDTMQ	10	
	CT681 P32 DE	AEGQLGDTMQIVSLQLNKMK	30	
	CT681 P30 E	IFDTTTLNPTIAGAGDVKAS	30	
	CT681 P31 E	IAGAGDVKASAEGQLGDTMQ	20	
	CT681 P27 F	MFTPYIGVKWSRASFDSDTI	40	
	**CT681 P28 F**	**SRASFDSDTIRIAQPRLVTP**	**60**	**70-80**
	CT681 P29 F	RIAQPRLVTPVVDITTLNPT	20	
	CT681 P30 F	VVDITTLNPTIAGCGSVAGA	40	
	CT681 P31 F	IAGCGSVAGANTEGQISDTMQ	20	
	CT681 P32 F	TEGQISDTMQIVSLQLNKMK	10	
	CT681 P27 G	MFTPYIGVKWSRASFDSNTI	20	
	**CT681 P28 G**	**SRASFDSNTIRIAQPKLAKP**	**50**	**75-85**
	CT681 P29 G	RIAQPKLAKPVVDITTLNPT	10	
	CT681 P30 G	VVDITTLNPTIAGCGSVVAA	0	
	CT681 P31 G	IAGCGSVVAANSEGQISDTMQ	10	
	CT681 P32 G	SEGQISDTMQIVSLQLNKMK	30	

Supplementary Table 1. **a)** Sequences of CTH522 overlapping peptides. Peptides in bold: recognized by 50% or more of the CTH522 vaccinated participants. b) Frequency of CTH522 vaccinated participants (n=10) with a peptide response >170 pg/ml (cut-off based on peptide responses in the placebo group). **c)** based on SvA2497, SvB/HAR-36, SvC/TW-3, SvD/UW-3/Cx, SvE Bour, SvF/IC-Cal-3, SvG/11222, SvH/UW4, SvI/UW-12, SvIa/IU-4168, SvJ/UW36/Cx, SvJa/IUA795, SvK/UW31.

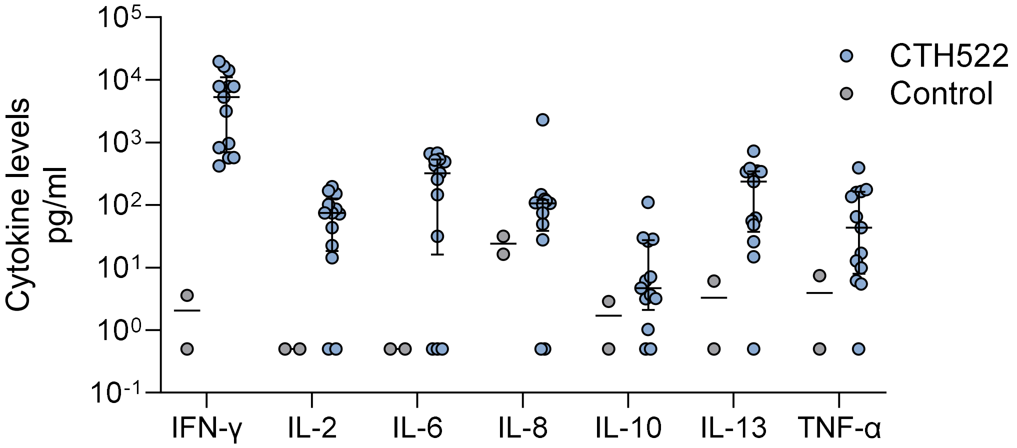

Supplementary Figure 1**. T cell responses after CTH522/CAF®01 immunization in humans.** CTH522/CAF®01 vaccinated and placebo participants. PBMC samples from CTH522 vaccinated (n=13) and placebo samples (n=2) were stimulated *in vitro* with 5 µg/ml of CTH522 for 5 days. Cytokine responses were measured in harvested supernatants diluted 1:2 by a MSD V-plex kit (Meso Scale Discovery, Proinflammatory panel 1 kit). Standard and samples were measured in duplicate. Data points represent mean cytokine levels in antigen stimulated wells after subtraction of media only and are for the CTH522 vaccinated group lines represent median with 25th and 75th percentiles, and for the control group lines show the median. Source data are provided as a Source Data file.

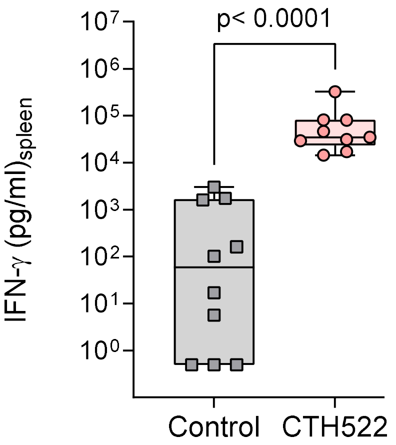

Supplementary Figure 2. **Recognition of UV-inactivated *C.t.* SvD after CTH522 vaccination**. Female B6C3F1 mice were immunized three times s.c. with 10 µg CTH522/CAF®01 or Sham-immunized. Two weeks post 3rd immunization individual splenocytes were isolated and stimulated *in vitro* with 5 µg/ml UV-inactivated *C.t.* SvD. IFN-γ responses were measured in supernatants after 3 days by ELISA. Data points represent mean IFN-γ response of triplicate readings after subtraction of media only. Graphs represent a pool of two individual experiments (CTH522 vaccinated group n=9, Control group n=10) and are presented as a Box and Whiskers plot with median and 25th and 75th percentiles (Box) and Min to Max, all data points (Whiskers). A two tailed Mann-Whitney U test was used for comparison among groups. Source data are provided as a Source Data file.

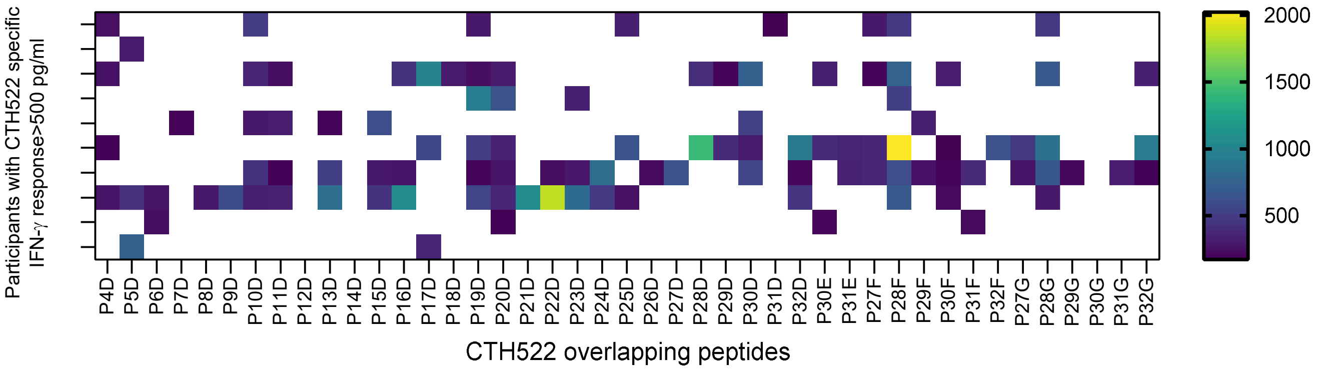

Supplementary Figure 3. **Heatmap of human T cells responses to CTH522**. Heatmap of peptide IFN-γ responses of CTH522 vaccinated participants with a total CTH522 specific IFN-γ response >500 pg/ml (n=10). IFN-γ responses were measured in supernatants after *in vitro* stimulation for 5 days with 20-21-mer peptides overlapping CTH522. Data represents median IFN-γ response of triplicate readings after subtraction of media only. Source data are provided as a Source Data file.

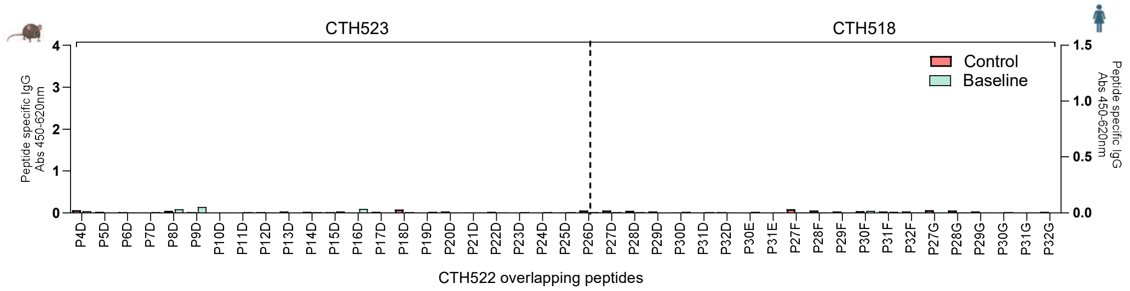

Supplementary Figure 4. **Peptide recognition in control sample**s. A pool of serum from 16 control B6C3F1 mice and 14 individual baseline human serum samples were used as negative control samples for the murine and human study, respectively Samples were diluted 1:200 and tested against the panel of overlapping CTH522 peptides (20-21-mers with 10 amino acid overlap). Mouse and human symbols were created from Biorender.com. Source data are provided as a Source Data file.

a

MHHHHHH**D**AISMRMGYYGDFVFDRVLKTDVNKEFQMGAAPTTKDIAGLENDPTTNVARPN
PAYGKHM**D**AISMRMGYYGDFVFDRVLKTDVNKEFQMGAAPTTKDIAGLENDPTTNVARPN
PAYGKHM**D**AISMRMGYYGDFVFDRVLKTDVNKEFQMGAAPTTKDIAGLENDPTTNVARPN
PAYGKHM**D**AISMRMGYYGDFVFDRVLKTDVNKEFQMGAAPTTKDIAGLENDPTTNVARPN
PAYGKHM

b

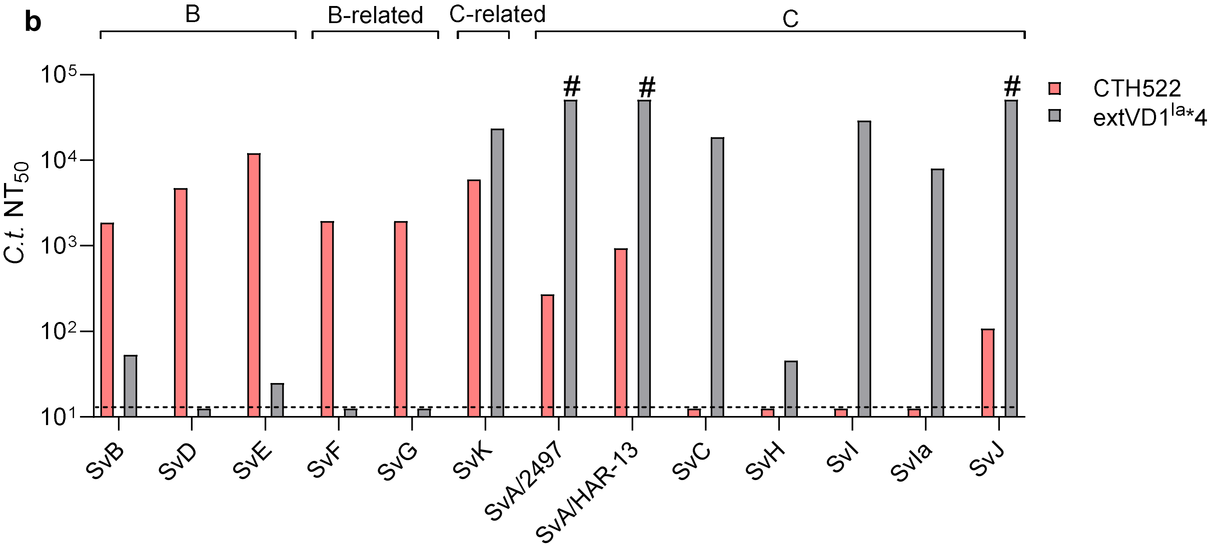

Supplementary Figure 5**. Neutralization of *C.t.* serovars with serum from mice vaccinated with CTH522 or a SvIa VD1 construct. a** Sequence of a homologous immuno-repeat construct extVD1^{Ia}*4. Based on the MOMP SvIa amino acid sequence with addition of six N-terminal histidines, a synthetic DNA construct was codon-optimized for expression in *E. coli* followed by insertion into the pJexpress 411 vector (ATUM, Newark, CA, USA). Purification was done essentially as described in Methods **b** *In vitro* neutralization of *C.t.* serovars with pools of serum samples from CTH522/CAF®01 and extVD1^{Ia}*4/CAF®01 vaccinated B6C3F1 (H-2b,k) and A/J (H-2a) mice, respectively. Bars represent the calculated mean NT_{50} of duplicate or triplicate readings. #, NT_{50} > 51200. Source data are provided as a Source Data file.

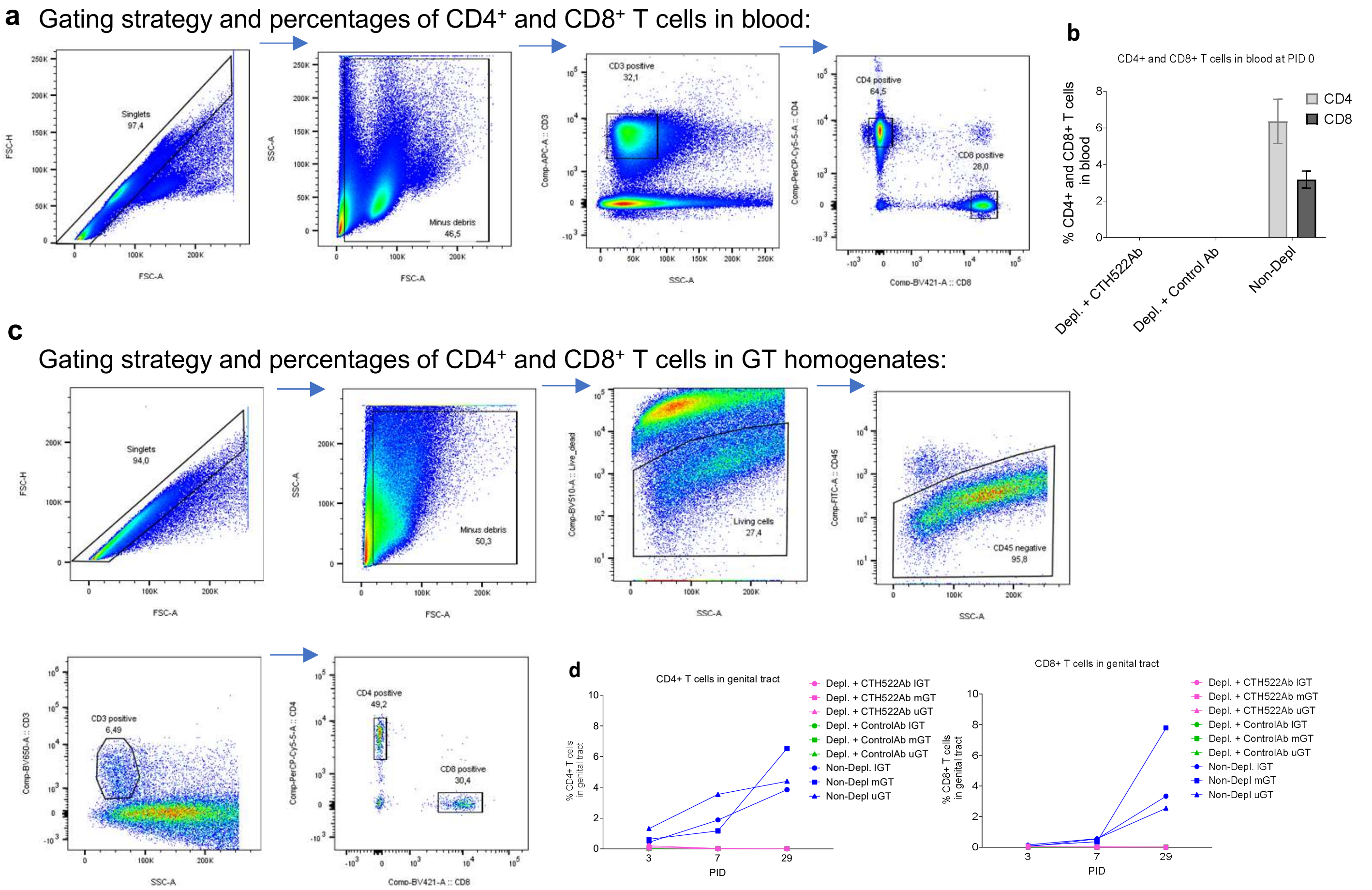

Supplementary Figure 6.**Gating strategies and prescence of CD4$^+$ and CD8$^+$ T cells.** **a** Gating strategy for partial bleed at PID0 in depleted and non-depleted C3H/HeN mice. **b** % CD4$^+$ and CD8$^+$ T cells in blood samples at PID0. Each bar depicts the mean±SD of the % of CD4 and CD8 T cells in 9 pools of 3 mice of each group. **c** Gating strategy for homogenates of genital tracts tissue. To differentiate cells located in the vasculature and parenchyma for FACS analysis, C3H/HeN mice were injected with anti-mouse CD45.2 FITC labelled (BD Pharmingen) antibodies. **d** % CD4$^+$ and CD8$^+$ T cells in lGT (lower), mGT (middle, Uterine horns) and uGT (upper, oviducts and ovaries) at different time points post infection. Data point represents mean CD4$^+$ and CD8$^+$ T cell percentage in the genital tract tissue of two pools of two mice per group, except in uGT at PID3 and 7 where one pool of 4 mice were analysed. Cells were stained with combinations of the following anti-mouse antibodies conjugated to fluorochromes (company, clone, dilution): FC Block (Biolegend, 2.4G2, 1:100), Viability-eFluor506 (eBioscience, #65-0866-14, 1:500), α-CD3-BV650 (Biolegend, 17A2, #100299, 1:200), α-CD3-APC (BD Bioscience, 145-2C11, #553066, 1:200) α-CD4-PerCP/Cy5.5 (BioLegend, RM4.4, #116012, 1:200), α-CD8-BV421 (Biolegend, 53-6.7, #100738, 1:200). The stained cells were analyzed using a Flow cytometer (BD LSRFortessa, BD Bioscience) and FlowJo Software. **d** Source data are provided as a Source Data file.

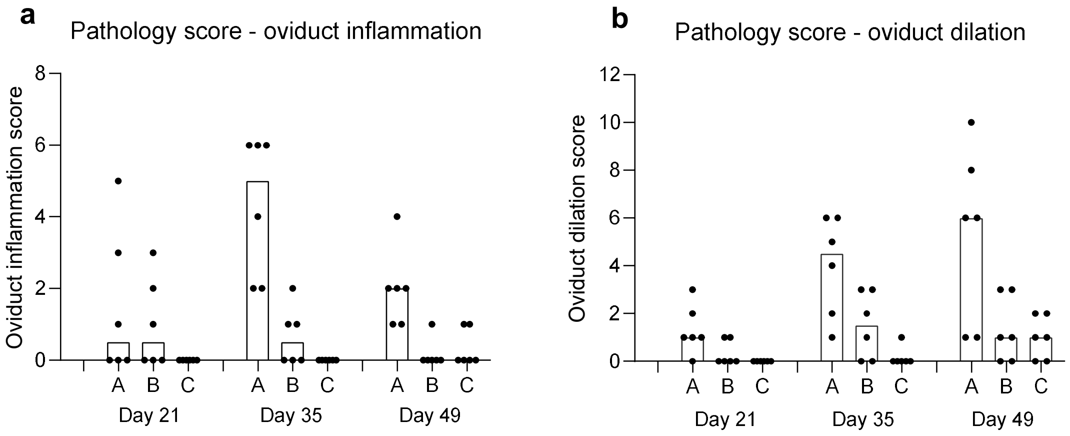

Supplementary Figure 7. **Pathology score in the oviducts**. **a** Oviduct inflammation was scored from 0-4, ranging from normal to extreme inflammation and **b** Oviduct dilation was scored from 0-5 ranging from no dilation to extreme dilation. Each dot represents the sum of right and left oviduct from one mouse. In some mice only one oviduct could be scored. A: CD4/CD8–depleted/*C.t.* SvD infected (n=6), B: non-depleted/*C.t.* SvD infected (n=6), C: non-depleted/non-infected at different time points post infection (n=6). Source data are provided as a Source Data file.

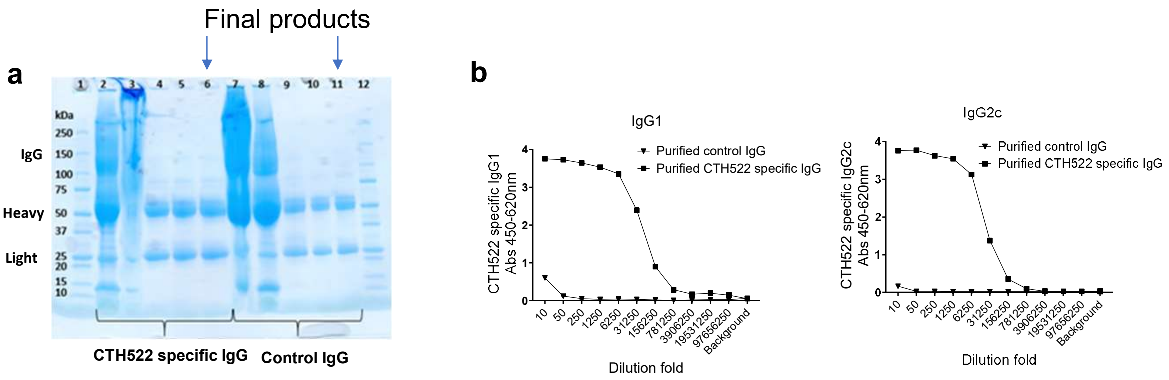

Supplementary figure 8**. IgG from CTH522/CAF01 vaccinated and control mice a** SDS page of purified IgG from CTH522-vaccinated and naïve mice (n=100). The purity of end product and intermediate steps were assessed by SDS-PAGE. Lane 1 and 12; molecular weight marker, marking whole IgG at 150 kDa, heavy chain of IgG at 50 kDa and light chain of IgG at 25 kDa. Lane 2 and 7; pool of serum from CTH522-vaccinated and control mice, respectively. Lane 3 and 8; negative Protein G column flow-through of serum from CTH522-vaccinated and naïve mice, respectively. Lane 4 and 9; eluate of purified antibodies from CTH522-vaccinated and naïve mice, respectively, before dialysis. Lane 5 and 10; eluate of purified IgG from CTH522-vaccinated and naïve mice, respectively, after dialysis. Lane 6 and 11: eluate of purified IgG from CTH522-vaccinated and naïve mice, respectively, after sterile filtration, thus the final product adoptively transferred to C3H/HeN mice **b** Subclass of CTH522-specific IgG. Purified IgG from control and CTH522-vaccinated mice were serial diluted, added to CTH522 antigen coated plates and captured by mouse IgG1 capture antibody and mouse IgG2c capture antibody and binding ability was analyzed by ELISA. Each sample was analyzed in triplicates and symbols represent the mean absorbance value with SEM at each titration step. **b** Source data are provided as a Source Data file.

a

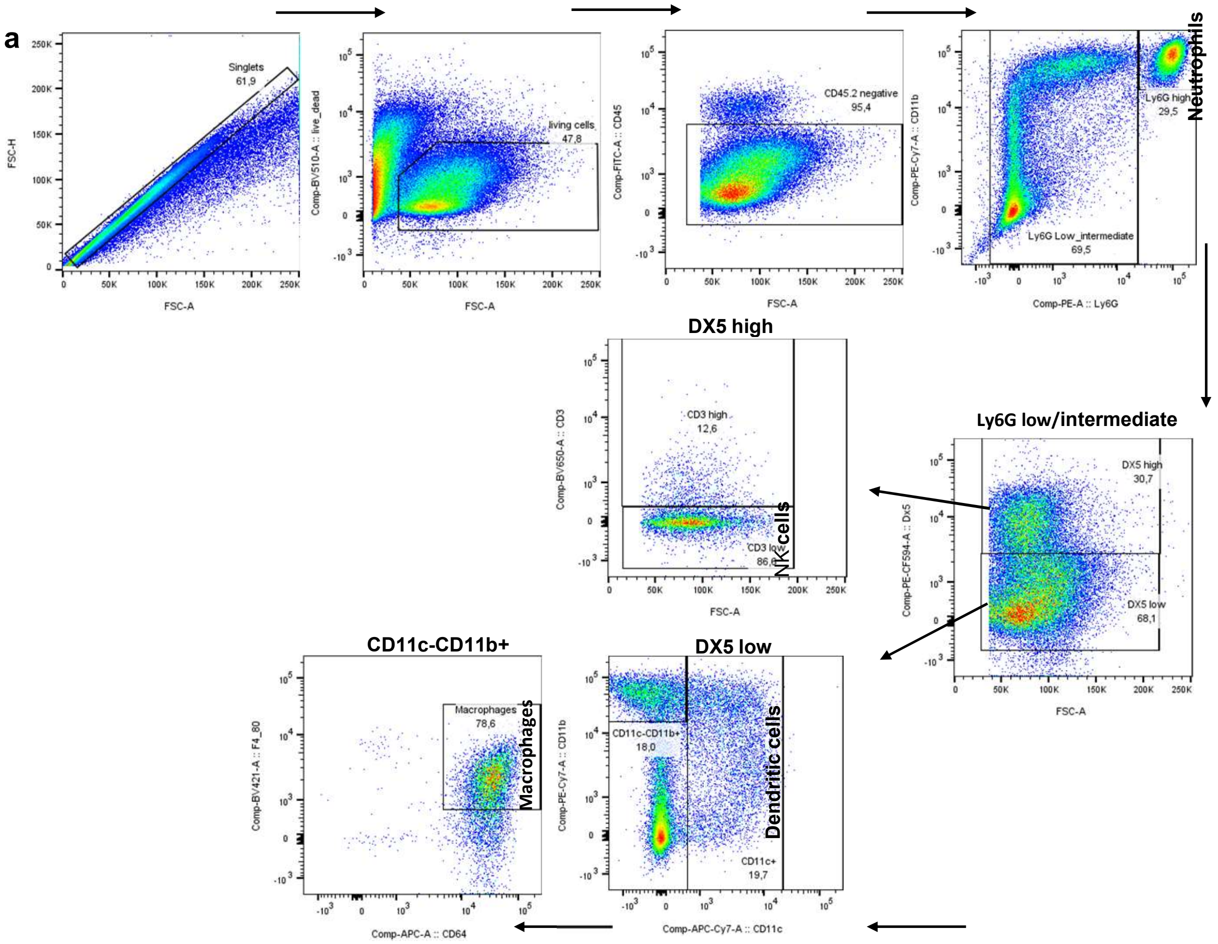

b

Groups	% cells in uGT			
	Neutrophils	Macrophages	NK cells	Dendritic cells
**Depl. + CTH522 IgG Pool 1**	4.6	2.8	25.3	10.5
**Depl. + CTH522 IgG Pool 2**	11.8	4.0	26.6	12.5
**Depl. + Control IgG Pool 1**	29.6	9.8	17.7	10.5
**Depl. + Control IgG Pool 2**	28.3	6.3	17.5	9.4

Supplementary Figure 9. **a** Gating strategy for homogenates of uGT (oviduct and ovaries) tissue at PID29. Two pools/group (n=2/pool) were analysed. To differentiate cells located in the vasculature and parenchyma for FACS analysis, C3H/HeN mice were injected with anti-mouse CD45.2 FITC labelled antibodies (BD Pharmingen, clone 104, cat. #560695BD). The GT tissue were incubated before homogenization for 1 hour at 37°C, 5% CO_2 in type IV collagenase (0.8 mg/ml) (Sigma) and 30 minutes in DNAse I (Roche) (0.08 mg/ml) and processed both before and after incubation with gentleMACS™ Dissociator (Miltenyi Biotec). Washed and centrifuged (700xg, 5 min.) cell pellets from all organs were resuspended in RPMI-1640 (Thermo Fisher Sci., Gibco, cat. #21875-034) supplemented with 1% (vol/vol) L-glutamine, 1% non-essential amino acids, 1% sodium pyruvate, 50 µM 2-mercaptoethanol, 1% penicillin-streptomycin, 1% HEPES and 10% heat-inactivated FBS (HI-FBS) (Biowest, South American origin, VWR) and further homogenized through a 100 µm nylon filter (Falcon). GTs cells were stained with combinations of the following anti-mouse antibodies conjugated to fluorochromes (company, clone, catalog, dilution): FC Block (Biolegend, 2.4G2, #553142 1:100), Viability-eFluor506 (eBioscience, #65-0866-14, 1:500), CD11b-PEcy7 (BD Bioscience, M1/70, #552850, 1:300), F4/80-BV421 (Biolegend, BM8, #123131, 1:200), CD11c-APC-Cy7 (Biolegend, N418, #117324, 1:100), Ly6G-PE (BD Bioscience, 1A8, #551461, 1:00), CD64-APC (Biolegend, X54-5/7.1, #139305, 1:100), CD49b-PE-CF594 (Biolegend, DX5, #108924, 1:100), CD3e-BV650 (Biolegend, 17A2, #100229,1:200). The stained cells were analyzed using a Flow cytometer (BD LSRFortessa, BD Bioscience) and FlowJo Software. **b** % cells in uGT homogenates out of CD45.2 negative cells.

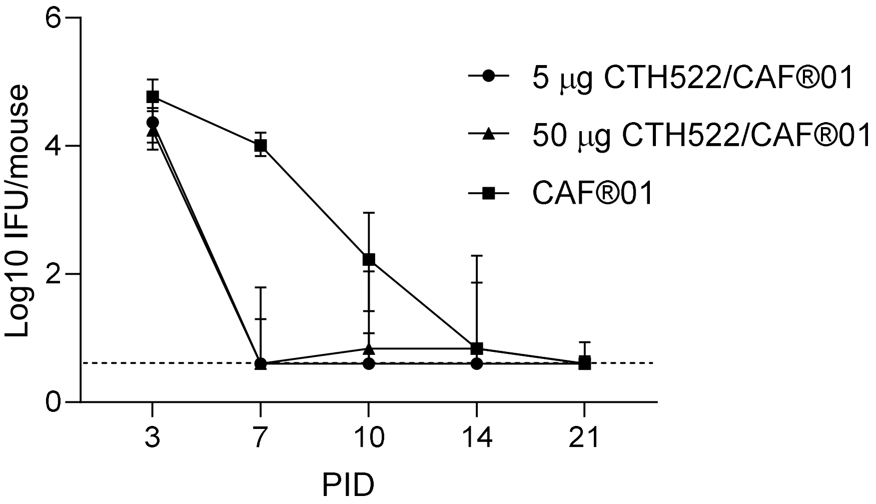

Supplementary Figure 10. **Protection 1 year post vaccination**. B6C3F1 mice were immunized two times s.c. with 5µg or 50µg CTH522/CAF®01 with a 2 week interval and boosted intranasally with 10µg CTH522. Mice immunized with CAF®01 were included as controls. One year post 3rd vaccination mice were challenged i.vag. with 1 x 10^5 IFU/mouse of *C.t.* SvD. IFU were recovered from vaginal swabs at PID3, 7, 10, 14, and 21 (n=6) and presented as median Log10 IFU/mouse with 25th and 75th percentiles for each group. Source data are provided as a Source Data file.

a Gating strategy for Cytokine+ CD4 T cells (Splenocytes)

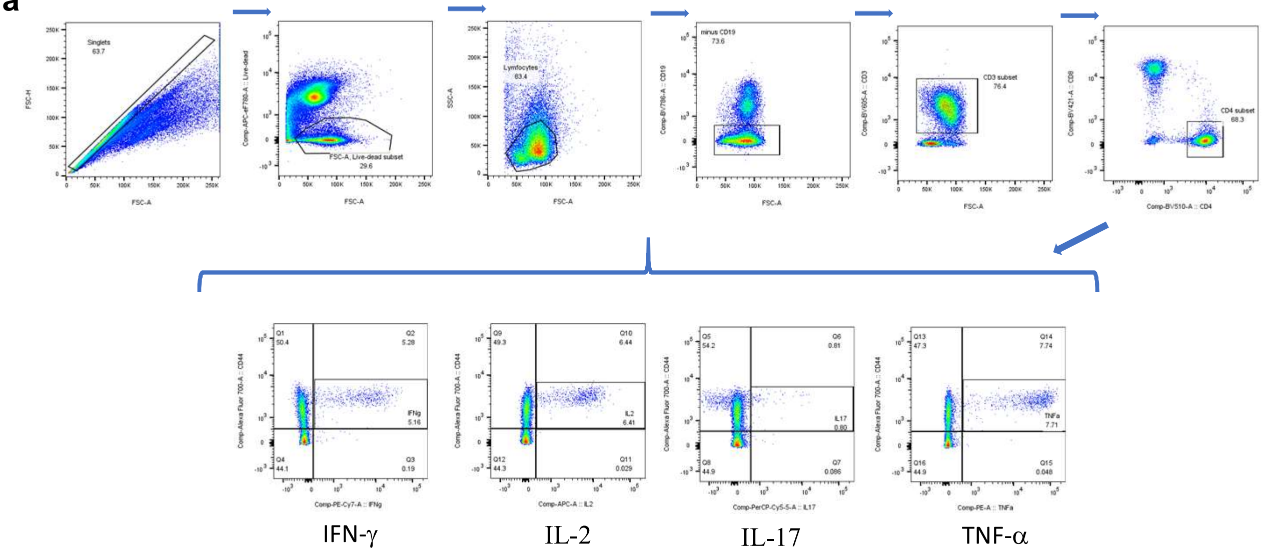

b Gating strategy for Cytokine+ CD4 T cells (Genital tract tissue)

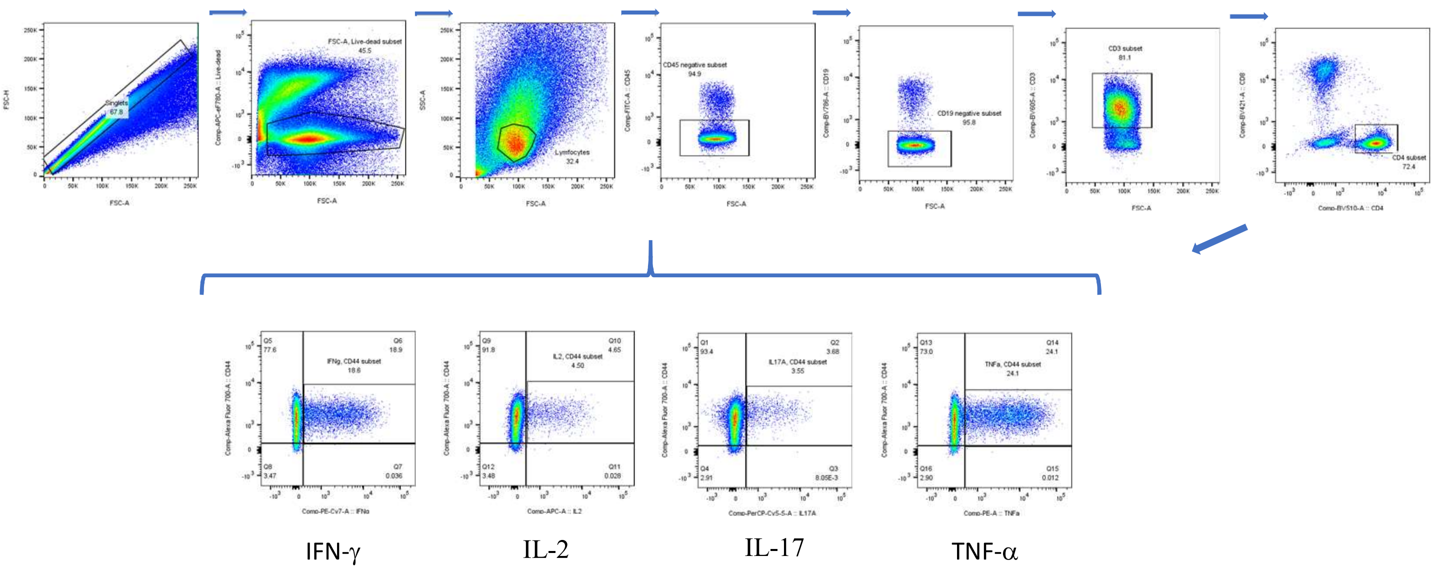

Supplementary Figure 11**.** Gating strategies for cytokine+ CD4$^+$ T cells in **a** Splenocytes and **b** Genital tract tissue. See "Methods" section for further details.

PROTOCOL

A phase I first in human, double-blind, parallel, randomised and placebo controlled clinical trial of the safety of SSI's adjuvanted chlamydia vaccine CTH522 in healthy women aged 18 to 45 years

Trial code: CHLM-01

EudraCT number: 2015-004330-10

Trial phase: I

Final 5.0

Clinical trial manager:

[redacted]

Vaccine Development Department,

Statens Serum Institut

Denmark

[redacted]

1 Synopsis

**Name of sponsor:**<br>Statens Serum Institut		**Chlamydia vaccine CTH522**
**Product name:**<br>**CTH522**		**Final 5.0**
**Title of the trial:**	A phase I first in human, double-blind, parallel, randomised and placebo controlled clinical trial of the safety of SSI's adjuvanted chlamydia vaccine CTH522 in healthy women aged 18 to 45 years.	
**Investigators and Investigational Sites:**	Dr. [redacted], Imperial College, London, United Kingdom – Principal Investigator<br>Dr. [redacted], Imperial College, London, United Kingdom – Co-investigator	
**Country:**	United Kingdom.	
**Trial period:**	Q2 2016 to Q1 2017	
**Trial code / EUDRACT number:**	2015-004330-10	
**Clinicaltrials.gov identifier**	NCT02787109	
**Trial phase:**	Phase I – first in human	
**Trial objectives:**	**Primary Objectives:**<br>• To evaluate the safety of CTH522-CAF01 adjuvanted chlamydia vaccine when administered intramuscularly<br>• To evaluate the safety of CTH522-Al$(OH)_3$ adjuvanted chlamydia vaccine when administered intramuscularly<br>• To evaluate the safety of CTH522 chlamydia vaccine when administered intranasally in individuals vaccinated (intramuscularly) with adjuvanted CTH522<br>• Evaluation of adverse events/reactions and laboratory safety<br>**Secondary Objectives:**<br>• To evaluate the serum IgG antibody responses after vaccination with CTH522<br>**Exploratory objective:**<br>• To evaluate the systemic and mucosal immunogenicity of CTH522-CAF01 and CTH522-Al$(OH)_3$ following the trial vaccination schedules	
**Trial endpoints:**	**Primary endpoints - safety:**<br>• Solicited local injection site reactions (recorded at any visit) after intramuscular (IM) administration (pain, erythema, tenderness, pruritus, warmth, stiffness and swelling)<br>• Solicited local reactions (recorded at any visit) after IN administration (discharge, including bleeding, congestion, discomfort, sneezing and cough)<br>• Solicited systemic reactions (recorded at any visit) after IM and IN administration (abnormally raised temperature, chills, myalgia, malaise, fatigue, rash, headache, nausea and vomiting, and clinically significant abnormal values among full blood count, liver function test and renal profile results)	

**Name of sponsor:** Statens Serum Institut<br><br>**Product name:** CTH522	**Chlamydia vaccine CTH522**<br><br>**Final 5.0**
	**Secondary endpoint - immunology**<br>• Percentage of subjects achieving seroconversion for anti-CTH522 IgG antibody at any time points after IM vaccination(s)
	**Exploratory immunology:**<br>Immunogenicity against CTH522 as measured by<br>• Changes in values from baseline in:<br>○ Systemic, nasal, ocular and vaginal antibodies<br>○ Cell-mediated immune response as measured by Elispot and/or flow cytometry<br>○ Antibody responses measured by B-cell Elispot<br>○ Serum neutralising antibodies against serovars D-G<br>• Isolation and characterisation of CTH522–antigen specific memory B cells in the systemic compartments (dependent on the elicited specific memory B-cell numbers)
**Trial design:**	This clinical trial is a first in human trial comparing: (see table below)

Group	No of subjects	Day 0	Day 28	Day 112	Day 126	Day 140
		IM	IM	IM	IN	IN
**CTH522-CAF01**	15 F	CTH522 85 µg CAF01	CTH522 85 µg CAF01	CTH522 85 µg CAF01	CTH522 2X30 µg	CTH522 2X30 µg
**CTH522-$Al(OH)_3$**	15 F	CTH522 85 µg $Al(OH)_3$	CTH522 85 µg $Al(OH)_3$	CTH522 85 µg $Al(OH)_3$	CTH522 2X30 µg	CTH522 2X30 µg
**Placebo**	5 F	NaCl	NaCl	NaCl	NaCl	NaCl

The present trial is a phase I first in human, double blind, parallel and placebo controlled trial of SSI's adjuvanted chlamydia vaccine CTH522 (CTH522-CAF01 and CTH522-$Al(OH)_3$). The trial will be conducted at Imperial College Research site in UK.
Subjects are randomly assigned to one of the following three treatment groups in a ratio of 3:3:1. This trial consisted of 10 visits and 5 telephonic interviews.

**Trial population and number of subjects:**	Healthy female volunteers aged 18 to 45 years with no previous or present history of *Chlamydia trachomatis* infection. 15 volunteers will be included in the CAF01 group, 15 in the $Al(OH)_3$ group and 5 volunteers in the placebo group - i.e. up to 35 volunteers in total.

**Name of sponsor:** Statens Serum Institut		**Chlamydia vaccine CTH522**
**Product name: CTH522**		**Final 5.0**
**Inclusion criteria:**	1. Is a healthy female between 18 and 45 years of age on the day of first trial vaccination<br>2. Has provided signed informed consent<br>3. Is willing and likely to comply with the trial procedures<br>4. Is prepared to grant authorised persons access to their medical record<br>5. Willing to use acceptable contraceptive measures* during the trial (2 weeks before and 2 weeks after the trial)<br>*Heterosexually active female capable of becoming pregnant must (in addition to requiring male partner to use condoms) agree to use hormonal contraception, or to complete abstinence, from at least 2 weeks before the first vaccination until at least 2 weeks after the last. (Note: Periodic abstinence [e.g. calendar, ovulation, symptothermal, post-ovulation methods] and withdrawal and intrauterine device or intrauterine hormone-releasing system and progestogen-only oral hormonal contraception, where inhibition of ovulation is not the primary mode of action, are not acceptable methods of contraception)	
**Exclusion criteria:**	1. Has confirmed history of Pelvic Inflammatory Disease or significant gynaecological diseases<br>2. Is positive for *C. trachomatis* (PCR)<br>3. Is positive for gonorrhoea (urine), HIV, Hepatitis B/C, syphilis (blood)<br>4. Has a positive pregnancy test<br>5. Has a significant active disease - such as cardiac, liver, immunological, neurological, psychiatric; or clinically significant abnormality of haematological or biochemical parameters.<br>6. Has BMI of 35 kg/m^2 or greater<br>7. Is currently participating in another clinical trial with an investigational or non-investigational drug or device<br>8. Has received, or plans to receive, an active immunisation within 14 days of the start of the trial or any of the immunisation visits in this trial<br>9. Is currently receiving treatment with immunosuppressive agents e.g. oral, inhaled, nasal or injected corticosteroids. (Topical steroids are allowed, unless applied to the IM injection site.)<br>10. Is using an intrauterine device<br>11. Has a condition which in the opinion of the investigator is not suitable for participation in the trial<br>12. Known or confirmed allergy to any of the vaccine constituents	
**Investigational medicinal products:**	**Investigational products:**<br>• CAF01 group: 85 µg CTH522 with 625 µg/125 µg CAF01 in a total volume of 0.6 ml<br>• $Al(OH)_3$ group: 85 µg CTH522 with 0.425 mg $Al(OH)_3$ in a total volume of 0.6 ml<br>• 85 µg CTH522 chlamydia antigen for IN administration<br>• Placebo for IM and IN administration – i.e. saline	
**Dosage and route of administration:**	Dosages: 85 µg CTH522 with either 625 µg/125 µg CAF01 or 0.43 mg $Al(OH)_3$ for IM administration and 60 µg unadjuvanted CTH522 for IN administration. Routes of administration: Three IM injections of adjuvanted vaccine followed by two IN administration of un-adjuvanted vaccine	

**Name of sponsor:** Statens Serum Institut<br>**Product name:** **CTH522**		**Chlamydia vaccine CTH522**<br>**Final 5.0**
**Statistical Methods:**	Baseline is defined as the last assessment with available data prior to the first administration of trial medication.<br>Categorical data will be summarised descriptively by treatment, using number and percentages of subjects. Continuous data will be presented using the number of subjects (n), mean, standard deviation (SD), median, lower quartile, upper quartile, minimum and maximum. Both the absolute values and the change from baseline will be presented.<br>Descriptive statistics for all endpoints will be presented by treatment group and visit (if applicable).<br>All statistical tests will be carried out as two-sided and performed on a 5 % significance level unless otherwise stated. Estimated treatment differences and 95 % CIs will be presented together with the corresponding p-value.	

2 Table of contents

1 Synopsis **2**

2 Table of contents **6**

3 List of abbreviations and definitions **9**

4 Signature page(s) **11**

5 Relevant addresses **12**

6 Introduction **15**

7 Trial objectives **17**

7.1 Primary objectives 17

7.2 Secondary objective 17

7.3 Exploratory objective 17

7.4 Endpoints 17

7.4.1 Primary endpoints - safety 17

7.4.2 Secondary endpoint - immunology 17

7.4.3 Exploratory endpoints – immunology 18

8 Investigational plan **19**

8.1 Overall design 19

8.2 Trial procedures 21

8.3 Trial population 27

8.4 Inclusion criteria 27

8.5 Exclusion criteria 28

8.6 Predetermined reasons for discontinuation 28

8.7 Temporary contraindications 29

8.8 Investigational events 30

8.8.1 Immunogenicity assessments 30

8.8.2 Safety assessments 31

8.8.3 Collection and handling of samples 31

8.9 Laboratory assays 32

8.10 Time schedule and recruitment 33

9 Investigational medicinal products **34**

9.1 Treatments administered 34

9.2 Doses and administration 34

9.2.1 Doses 34
9.2.2 Administration 35
9.3 Composition of trial vaccines when reconstituted 36
9.4 Packaging and labelling..37
9.5 Storage information..37
9.6 Transport of IMP..38
9.7 Randomisation procedure 38
9.8 Blinding and unblinding procedure..38
9.9 Treatment compliance 40
9.10 Drug accountability 40
9.11 Precautions and overdosing 40
9.12 Concomitant medication..40
9.13 Drug interactions..41

10 Ethical aspects 42

10.1 Risks and inconveniences 42
10.2 Benefits..43
10.3 Overall conclusion risks and benefits:..43

11 Adverse events 44

11.1 Definition and terms..44
11.2 Collection and Recording of AEs and SAEs 45
11.2.1 Collection..45
11.2.2 Recording..45
11.2.3 Assessments 46
11.3 Expedited reporting of adverse event 47
11.4 Data safety monitoring board 48

12 Trial Steering Committee...50

13 Data management and statistical analysis 51

13.1 General considerations 51
13.2 Data management..51
13.3 Clean file procedures..51
13.4 Coding of AEs and concomitant medication: 52
13.5 Analysis populations 52
13.6 Statistical methods 52
13.6.1 Demographics and other baseline characteristics..52

13.6.2 Safety analysis 53
13.6.3 Immunogenicity analysis 53
13.7 Sample size determinations 53
13.8 Interim analysis 54
13.9 Handling of missing data 54
13.10 Multiplicity 54
14 Good clinical practice considerations 55
14.1 ICH GCP / Declaration of Helsinki 55
14.2 Subject information and informed consent 55
14.2.1 General procedures for obtaining informed consent 55
14.3 Ethics committee submission and approval 56
14.4 Competent authority submission and approval 56
14.5 Subject data protection 56
14.6 Investigator's responsibilities 57
14.7 Curricula vitae 57
14.8 Indemnity statement 57
14.9 Training 57
14.10 Monitoring 58
14.11 Audit and inspection 58
14.12 Definition of source data 58
14.13 Archiving of essential documents 59
15 Agreement and financial settlement 61
16 Budget 62
17 Insurance 63
18 Confidentiality and disclosure 64
19 Protocol amendments 65
20 References 66
21 Appendices 68

3 List of abbreviations and definitions

AE	Adverse event
ADR	Adverse Drug Reaction
AIDS	Acquired immune deficiency syndrome
ALT	Alanine transaminase
AR	Adverse reaction
AST	Aspartate transaminase
BP	Blood pressure
CA	Competent authority
CI	Confidence interval
CIOMS	Council for International Organisations of Medical Sciences
CRC	Clinical research centre
CRF	Case report form, in this trial an electronic CRF (eCRF) is used
CRO	Contract research organisation
CTA	Clinical trial authorisation
CTR	Clinical trial report
CV	Curriculum vitae
DSMB	Data safety monitoring board
DSUR	Development safety update report
EC	Ethics committee
eCRF	Electronic case report form
EDC	Electronic data capture (system used for electronic recording in the eCRF)
EDTA	Ethylenediaminetetraacetic acid
ELISA	Enzyme-linked immunosorbent assay
Elispot	Enzyme linked immunospot assay
EMA	European Medicines Agency
EVCTM	Eudravigilance clinical trial module
FAS	Full analysis set
FDA	Food and Drug Administration
FSFV	First subject’s first visit
GCP	Good clinical practice according to ICH GCP E6
GMP	Good manufacturing practice
Hb	Haemaglobin
HepB	Hepatitis B vaccine
HCG	Human chorionic gonadotropin
HR	Heart rate
IB	Investigator’s brochure
ICH	International Conference on Harmonisation
ICF	Informed consent form
ICS	Intracellular cytokine staining

IF	Investigator's file
IFN-γ	Interferon gamma
IgA	Immunoglobulin A
IgG	Immunoglobulin G
IM	Intramuscular
IMP	Investigational medicinal product
IMPD	Investigational medicinal product dossier
IN	Intranasal
ITT	Intention-to-treat
i.v.	Intravenous
[redacted]	[redacted]
LLOQ	Lower limit of quantification
LLT	MedDRA low level term
LSLV	Last subject's last visit
MedDRA	Medical Dictionary for Regulatory Activities
MHRA	Medicines and Healthcare Products Regulatory Agency
PI	Principal investigator
p.o.	Per os
PBMC	Peripheral blood mononuclear cell(s)
PCR	Polymerase chain reaction
PP	Per protocol set
PT	MedDRA preferred term
QP	Qualified person
RBC	Red blood cells
RPM	Rotations per minute
SAE	Serious adverse event
SAP	Statistical analysis plan
SAS	Statistical analysis system
SD	Standard deviation
SDV	Source data verification
SmPC	Summary of product characteristics
SOP	Standard operating procedure
SSI	Statens Serum Institut, Denmark
STD	Sexually transmitted disease
SUSAR	Suspected unexpected serious adverse reaction
TFLs	Tables, figures and listings produced by the statistician
TMF	Trial master file
Treatment emergent	An event that emerges during treatment having been absent pre-treatment, or worsens relative to the pre-treatment state (ICH definition)
WBC	White blood cell
WHO	World Health Organisation
WHO-DDE	World Health Organisation Drug Dictionary Enhanced

4 Signature page(s)

Clinical trial manager, SSI:	[redacted]	(date & signature)
Non-clinical investigator:	[redacted]	(date & signature)
Principal investigator	[redacted]	(date & signature)
Trial statistician:	[redacted]	(date & signature)
Medically responsible, SSI:	[redacted]	(date & signature)
Pharmacovigilance – Safety Officer, SSI:	[redacted]	(date & signature)
Director of Vaccine Development Department & sponsor's representative, SSI:	[redacted]	(date & signature)

5 Relevant addresses

Principal Investigator: [redacted],
[redacted]

[redacted]

Non-clinical Investigator: [redacted]

[redacted]

Clinical trial manager, SSI: [redacted]

[redacted] [redacted]

Medically responsible, SSI: [redacted]

[redacted] [redacted]

Director Drug Safety and Medical Affairs, JPM:	[redacted]	[redacted]
Safety Officer, SSI:	[redacted]	[redacted]
Director, Vaccine Development, SSI and sponsor's representative:	[redacted]	[redacted]
Trial statistician:	[redacted]	[redacted]
Data manager:	[redacted],<br>[redacted]	[redacted]

Emergency telephone numbers and email address:

**Medically responsible, SSI:**	[redacted]	[redacted]
[redacted]	[redacted]	[redacted]
**Clinical trial manager, SSI:**	[redacted]	[redacted]
[redacted]	[redacted]	[redacted]
**Trial monitor, SSI:**	[redacted]	[redacted]
[redacted]	[redacted]	[redacted]
**Serious adverse event notification to [redacted] and SSI:**	[redacted]	[redacted]
		[redacted]
	[redacted]	[redacted]
	[redacted]	[redacted]
**Emergency phone numbers for subjects**	[redacted]	
	[redacted]	

6 Introduction

Worldwide, there are approximately 105.7 million new cases of Chlamydia each year. In spite of the availability of effective antimicrobial therapy, infection with *Chlamydia trachomatis (C. trachomatis)* is a public health concern because of its prevalence and potentially devastating reproductive consequences. In women the infection can lead to chronic pelvic pain, infertility, and potentially fatal ectopic pregnancy. In pregnant women, untreated chlamydia has been associated with pre-term delivery, as well as conjunctivitis and pneumonia in the newborn. Men develop urethritis and in rare cases epididymitis. Both sexes can develop reactive arthritis following genital infection.

Classical control measures such as diagnosis, screening programs, and treatment have been intensified in the recent years but the number of *C. trachomatis* infections is still increasing. As these infections are not effectively controlled, an effective vaccine is required (1, 2).

Moreover, the lack of evidence on the long-term effects of screenings programmes on reproductive sequelae may question whether such programmes are adequately effective (3). The use of screening programmes may improve the management of *C. trachomatis* infections, but the impact of these screening programmes on the risk of infertility may be limited as women who are at the highest risk might not seek medical consultation properly and/or timely. Thus, it is an international priority to develop a protective vaccine to combat the infection.

In the context of Chlamydia vaccine development it is becoming increasingly clear that both neutralising antibodies and CD4 Th1 cells are required to control infection (4, 5). *C. trachomatis* furthermore infects through the genital tract and recovery of bacteria from the cervix inversely correlates with secretory IgA titres, thus IgA could provide an important additional layer of first line defence (6).

SSI has developed a molecular designed novel multivalent vaccine antigen (CTH522). It is a recombinant version of the major outer membrane molecule (MOMP) from *C. trachomatis,* designed to induce broadly neutralizing antibodies. CTH522 is highly immunogenic and contains conserved T cell epitopes recognized which are frequently by *C. trachomatis* infected patients and is linked to an optimized module that contains the main neutralizing epitope from the 4 most frequent human *C. trachomatis* serovars (5, 7).

Aluminium salts are by far the most widely used adjuvant in vaccines to date. Their mechanism of action is not yet fully understood. It is known, however, that they support the development of antibodies but not optimally support the development of Th1 cells, which are thought to play a key role in obtaining protective immunity against *C. trachomatis* (8, 9). This has prompted the development of new adjuvants at SSI, such as the adjuvant CAF01.

The CAF01 adjuvant promotes both strong neutralising antibody titres and Th1 responses in mice, pigs and non-human primates (5, 10, 11). Furthermore, CAF01 has proven safe and immunogenic in clinical trials (12).

Most mucosal compartments of the body have local mucosal immune inductive sites. However, as the genital tract lacks these immune inductive sites, it is important to develop an alternative immunization strategy that utilizes other mucosal inductive sites to promote local genital tract immunity. It has been widely documented that intranasal (IN) immunization can induce mucosal immunity in both the respiratory and genital tract (13). Recently, studies in mice and pigs have shown that prime-boost regimes using CAF01 can facilitate induction of mucosal IgA in the genital tract (11).

CTH522 protects against genital *C. trachomatis* infection in the mouse model and is highly immunogenic in both mice, rabbits and pigs (Olsen JID, preclinical data). There was no evidence of a broader toxic effect of CTH522-CAF01 and CTH522-Al$(OH)_3$, when administered IM and boosted IN with un-adjuvanted CTH522 to minipigs in a more intensive regimen than anticipated in the clinical program. All non-clinical information supports the suitability of CTH522-CAF01 and CTH522-Al$(OH)_3$ as a safe, immunogenic, and possibly effective vaccine to induce immunity in *C. trachomatis* uninfected individuals.

On the basis of the results of these studies, it is considered safe to enter a clinical phase I with CTH522-CAF01 and CTH522-Al$(OH)_3$ vaccine including 35 healthy female between 18 and 45 years. The overall aim of this Phase I, first in human clinical trial is to investigate the safety and immunogenicity of a new chlamydia vaccine developed by SSI.

7 Trial objectives

7.1 Primary objectives

- To evaluate the safety of CTH522-CAF01 adjuvanted chlamydia vaccine when administered intramuscularly
- To evaluate the safety of CTH522-$Al(OH)_3$ adjuvanted chlamydia vaccine when administered intramuscularly
- To evaluate the safety of CTH522 chlamydia vaccine when administered intranasally in individuals vaccinated (intramuscularly) with adjuvanted CTH522

Safety evaluation comprises evaluation of adverse events/reactions and safety laboratory tests.

7.2 Secondary objective

- To evaluate the serum IgG antibody responses after vaccination with CTH522

7.3 Exploratory objective

- To evaluate the systemic and mucosal immunogenicity of CTH522-CAF01 and CTH522-$Al(OH)_3$ following the trial vaccination schedules

7.4 Endpoints

7.4.1 Primary endpoints - safety

- Solicited local injection site reactions (recorded at any visit) after intramuscular (IM) administration (pain, erythema, tenderness, pruritus, warmth, stiffness and swelling)
- Solicited local reactions (recorded at any visit) after IN administration (discharge, including bleeding, congestion, discomfort, sneezing and cough)
- Solicited systemic reactions (recorded at any visit) after IM and IN administration (abnormally raised temperature (> 38.3°C), chills, myalgia, malaise, fatigue, rash, headache, nausea and vomiting, and clinically significant abnormal values among full blood count, liver function test and renal profile results)

7.4.2 Secondary endpoint - immunology

- Percentage of subjects achieving seroconversion for anti-CTH522 IgG at any time point after IM vaccination(s) (see Section 13.6.3)

7.4.3 Exploratory endpoints – immunology

Immunogenicity against CTH522 as measured by

- Changes in values from baseline in:
    - Systemic, nasal, ocular and vaginal antibodies
    - Cell-mediated immune response as measured by Elispot and/or flow cytometry
    - Antibody responses measured by B-cell Elispot
    - Serum neutralising antibodies against serovars D-G
- Isolation and characterisation of CTH522–antigen specific memory B cells in the systemic compartments (dependent on the elicited specific memory B-cell numbers)

8 Investigational plan

8.1 Overall design

The present trial is a phase I first in human, double blind, parallel, randomised, and placebo controlled trial of SSI's adjuvanted chlamydia vaccine CTH522 (CTH522-CAF01 and CTH522-$Al(OH)_3$). The trial is conducted at Imperial College Research site in UK.

Subjects are randomly assigned to one of the following three treatment groups in a ratio of 3:3:1:

- CTH522-CAF01
- CTH522-$Al(OH)_3$
- Placebo

Table 8-1 Trial groups

Group	No of subjects	Day 0	Day 28	Day 112	Day 126	Day 140
		IM	IM	IM	IN	IN
**CTH522-CAF01**	15 F	CTH522 85 µg CAF01	CTH522 85 µg CAF01	CTH522 85 µg CAF01	CTH522 2X30 µg	CTH522 2X30 µg
**CTH522-$Al(OH)_3$**	15 F	CTH522 85 µg $Al(OH)_3$	CTH522 85 µg $Al(OH)_3$	CTH522 85 µg $Al(OH)_3$	CTH522 2X30 µg	CTH522 2X30 µg
**Placebo**	5 F	NaCl	NaCl	NaCl	NaCl	NaCl

This trial consists of 10 visits and 5 telephonic interviews (**Table 8-2**).

Table 8-2 Trial visits

**Visit – trial procedure**	**1**	**2**	**2a**	**3**	**4**	**4a**	**5**	**6**	**6a**	**7**	**7a**	**8**	**8a**	**9**	**10**
**Day**	**-90 to -1**	**0**	**3**	**14**	**28**	**31**	**42**	**112**	**115**	**126**	**129**	**140**	**143**	**154**	**168**
**Visit window (days)**	**0**	**0**	**±1**	**±2**	**±3**	**±1**	**±2**	**±7**	**±1**	**±2**	**±1**	**±2**	**±1**	**±2**	**±7**
Volunteer information and obtain informed consent	X														
Medical history, incl. medication and demography	X														
Inclusion/exclusion criteria	X	X													
Concomitant medication	X	X	X	X	X	X	X	X	X	X	X	X	X	X	X
Urinary Pregnancy test	X	X			X			X		X		X			X
Issue diary card	X														
Review diary card		X		X	X		X	X		X		X		X	X
General physical examination	X														X
Symptom directed physical examination		X		X	X		X	X		X		X		X	
Assessment of practical usability of soft cup (2)	X														
Height and weight (BMI) (3)	X														
Vital signs (BP, heart rate, oral temp.)	X	X		X	X		X	X		X		X		X	X
Laboratory safety testing	X	X		X	X		X	X		X		X		X	X
Randomisation		X													
Vaccine or placebo – IM (4)		X			X			X							
Vaccine or placebo – IN (4)										X		X			
Record AEs		X		X	X		X	X		X		X		X	X
Telephonic follow-up for AEs			X			X			X		X		X		
Vaginal soft cup, nasal and ocular strips		X			X			X		X		X		X	X
Test for *C. trachomatis* infection (PCR)	X														X
Screening for Gonorrhoea, Syphilis, Hepatitis B and C and HIV	X														

Visit – trial procedure	1	2	2a	3	4	4a	5	6	6a	7	7a	8	8a	9	10
Day	-90 to -1	0	3	14	28	31	42	112	115	126	129	140	143	154	168
Visit window (days)	0	0	±1	±2	±3	±1	±2	±7	±1	±2	±1	±2	±1	±2	±7
End of trial evaluation															X

Notes:

1. The timing of post-dose visits are set according to the immediately preceding dose; thus if a dose is delayed for any reason the subsequent visits are shifted accordingly.
2. Subjects who can't use soft cup will remain in the study but will not provide samples from soft cup for immunology testing
3. BMI will be calculated automatically in the eCRF based on height and weight entered
4. On dosing days, all procedures and assessments listed in Table 8-2 will be done before vaccination, including baseline vital signs. CTH522-CAF01 and CTH522-Al(OH_3) will be administered intramuscularly into the deltoid of the non-dominant arm. CTH522 will be administered intranasally into both nostrils. During administration of the IMPs resuscitation equipment will be immediately available for the management of anaphylaxis. The participant will stay for observation in case of immediate adverse events, which will be recorded. Vital signs will be taken 30 minutes after vaccination (+/- 10 minutes). For IM vaccinations, the injection site will be covered with a sterile dressing and removed after 30 minutes (+/- 10 minutes) and injection site inspected. Before the participant leaves, an oral thermometer, tape measure and diary card will be given, with instructions on use, along with the emergency telephone number to contact the research team if needed. Subjects in each group will be vaccinated only if there are no safety concerns raised 24 hours after the first subject in each group receives the first vaccination. For the first block (block size = 7) only one volunteer will be vaccinated per day.

8.2 Trial procedures

At visit 1 (screening visit, day -90 to -1): the subject's eligibility* will be assessed according to the pre-specified inclusion/exclusion criteria.

- Information on volunteers and obtain informed consent
- Medical history, incl. medication
- General physical examination including ENT, cardiovascular, pulmonary, neurological, gastrointestinal, urogenital, and dermatological systems, as well as injection sites
- Vital signs – including height, weight, blood pressure (BP), heart rate (HR) and oral temperature

- Test for *C. trachomatis* infection (PCR)
- Exclude subjects positive for gonorrhoea (urine), HIV, Hepatitis B/C, syphilis (blood)
- Blood samples for laboratory safety testing
- Pregnancy test (urine)
- Test for practical usability of soft cup
- Issue diary card

*Note: if the clinical site already has locally generated results of laboratory tests required at screening, and provided the sample(s) were collected within the screening window, the tests do not need to be repeated.

At visit 2 (inclusion and first IM vaccination, day 0):

- Review inclusion/exclusion criteria to decide on enrolment or not
- Review concomitant medication
- Review diary card for recording of safety and medication information. Review of diary cards for compliance
- Symptom directed physical examination
- Pregnancy test (urine)
- Vital signs - including BP, HR and oral temperature
- Blood sampling for laboratory safety testing
- Blood sampling for immunology testing
- Vaginal soft cup for immunology testing
- Ocular and nasal sampling for immunology testing
- Randomisation
- IM injection of vaccine or placebo
- Check for immediate adverse events (AEs) after 60 (+/- 10) minutes

<u>Visit 2a: a telephonic follow-up of safety, day 3 (+ 1):</u> trial staff will contact the subjects by phone to check for AEs and concomitant treatment, if relevant.

<u>At visit 3 (safety visit, day 14 [+ 2]):</u>

- Review concomitant medication
- Review of diary card
- Symptom directed physical examination
- Vital signs – including BP, HR and oral temperature
- Check for AEs since last visit
- Blood samples for laboratory safety testing
- Blood sampling for immunology testing

<u>At visit 4 (second IM vaccination, day 28 [+ 3]):</u>

- Review concomitant medication
- Review of diary card
- Symptom directed physical examination
- Pregnancy test (urine)
- Vital signs - including BP, HR and oral temperature
- Check for AEs since last visit
- Blood sampling for immunology testing
- Blood samples for laboratory safety testing
- <u>IM</u> injection of vaccine or placebo
- Check for immediate AEs after 60 (+/- 10) minutes
- Vaginal soft cup for immunology testing
- Ocular and nasal sampling for immunology testing

Visit 4a: a telephonic follow-up of safety, day 31 (+ 1): trial staff will contact the subjects by phone to check for AEs and concomitant treatment.

At visit 5 (safety visit, day 42 [+ 2]):

- Review concomitant medication
- Review of diary card
- Symptom directed physical examination,
- Vital signs - including BP, HR and oral temperature
- Check for AEs since last visit
- Blood samples for laboratory safety testing
- Blood sampling for immunology testing

At visit 6 (third IM vaccination, day 112 [+ 7]):

- Review concomitant medication
- Review of diary card
- Symptom directed physical examination
- Pregnancy test (urine)
- Vital signs - including BP, HR and oral temperature
- Check for AEs since last visit
- Blood sampling for laboratory safety testing
- Blood sampling for immunology testing
- IM injection of vaccine/placebo
- Check for immediate AEs after 60 (+/- 10) minutes
- Vaginal soft cup for immunology testing
- Ocular and nasal sampling for immunology testing

Visit 6a: a telephonic follow-up of safety, day 115 (+ 1): trial staff will contact the subjects by phone to check for AEs and concomitant treatment.

At visit 7 (first IN vaccination, day 126 [+ 2]):

- Review concomitant medication
- Review of diary card
- Check for AEs since last visit.
- Symptom directed physical examination
- Pregnancy test (urine)
- Vital signs - including BP, HR and oral temperature
- Blood samples for laboratory safety testing
- Blood sampling for immunology testing
- Ocular and nasal sampling for immunology testing
- Vaginal soft cup for immunology testing
- IN administration of vaccine or placebo
- Check for immediate AEs after 60 (+/- 10) minutes

Visit 7a: a telephonic follow-up of safety, day 129 (+ 1): trial staff will contact the subjects by phone to check for AEs and concomitant treatment.

At visit 8 (second IN vaccination, day 140 [+ 2]):

- Review concomitant medication
- Review of diary card
- Symptom directed physical examination
- Pregnancy test (urine)
- Vital signs - including BP, HR and oral temperature
- Check for AEs since last visit.

- Blood samples for laboratory safety testing
- Blood sampling for immunology testing
- Ocular and nasal sampling for immunology testing
- Vaginal soft cup for immunology testing
- IN administration of vaccine or placebo
- Check for immediate AEs after 60 (+/- 10) minutes

Visit 8a: a telephonic follow-up of safety, day 143 (+ 1): trial staff will contact the subjects by phone to check for AEs and concomitant treatment, if relevant.

At visit 9 (safety and immunology visit, day 154 [+ 2]):

- Review concomitant medication
- Review of diary card
- Symptom directed physical examination
- Vital signs - including BP, HR and oral temperature
- Check for AEs since last visit
- Blood samples for laboratory safety testing
- Blood sampling for immunology testing
- Ocular and nasal sampling for immunology testing
- Vaginal soft cup for immunology testing

At visit 10 (safety, immunology and end of trial visit, day 168 [+ 7]):

- Review concomitant medication
- Review of diary card
- Check for AEs since last visit
- General physical examination

- Pregnancy test (urine)
- Vital signs - including BP, HR and oral temperature
- Test for *C. trachomatis* infection (PCR)
- Blood samples for laboratory safety testing
- Blood sampling for immunology testing
- Ocular and nasal sampling for immunology testing
- Vaginal soft cup for immunology testing
- End of trial evaluation

Note: Early termination will include all the procedures/activities (as at Visit 10) at the early termination visit.

8.3 Trial population

This trial will enrol healthy female volunteers aged 18 to 45 years with no previous or present history of *C. trachomatis* infection. A total of 15 volunteers will be included in the CAF01 IM group, 15 in the $Al(OH)_3$ IM group and 5 volunteers in the Placebo (IM and IN) group - i.e. 35 volunteers in total.

8.4 Inclusion criteria

1. Healthy females between 18 and 45 years of age on the day of first trial vaccination
2. Has provided signed informed consent
3. Is willing and likely to comply with the trial procedures
4. Is prepared to grant authorised persons access to their medical record
5. Willing to use acceptable contraceptive measures* during the trial (2 weeks before and 2 weeks after the trial)

*Heterosexually active female capable of becoming pregnant must (in addition to requiring male partner to use condoms) agree to use hormonal contraception, or to complete abstinence, from at least 2 weeks before the first vaccination until at least 2 weeks after the last.
(Note: Periodic abstinence [e.g. calendar, ovulation, symptothermal, post-ovulation methods], withdrawal, intrauterine device or intrauterine hormone-releasing system, and progestogen-only

oral hormonal contraception, where inhibition of ovulation is not the primary mode of action, are not acceptable methods of contraception)

8.5 Exclusion criteria

1. Has confirmed history of Pelvic Inflammatory Disease or significant gynaecological diseases

2. Is positive for urine *C. trachomatis* (PCR)

3. Is positive for gonorrhoea (urine), HIV, Hepatitis B/C, syphilis (blood)

4. Has a positive urine pregnancy test

5. Has a significant active disease - such as cardiac, liver, immunological, neurological, psychiatric; or clinically significant abnormality of haematological or biochemical parameters

6. Has BMI of 35 kg/m^2 or greater

7. Is currently participating in another clinical trial with an investigational or non-investigational drug or device

8. Has received, or plans to receive, any immunisation within 14 days of the start of the trial or any of the immunisation visits in this trial

9. Is currently receiving treatment with immunosuppressive agents e.g. oral, inhaled, nasal or injected corticosteroids. (Topical steroids are allowed, unless applied to the IM injection site.)

10. Is using an intrauterine device

11. Has a condition which in the opinion of the investigator is not suitable for participation in the trial

12. Known or confirmed allergy to any of the vaccine constituents

8.6 Predetermined reasons for discontinuation

A subject is free to discontinue from the clinical trial at any time without giving a reason. The date and reason, if given, for discontinuation from the clinical trial must be recorded in the termination page of the eCRF.

The investigator may at any time withdraw a subject if her participation is no longer considered safe or relevant.

In case of pregnancy and/or anaphylactic reactions the subject must be withdrawn.

If a subject for any reason leaves or is withdrawn from the trial or has a significant protocol non-compliance she will be replaced by a new subject, at the discretion of the Sponsor and PI. However, replacements will not take place after 2nd IM vaccine/placebo at Visit 4. A maximum of 5 replacements per trial group will be allowed.

Because the healthy subjects gain no benefit from participation in the clinical trial, dosing in the trial must be stopped in case

- there is an SAE considered to be related to the IMP
- there are two or more severe AEs considered to be at least possibly related to the IMP (based on the judgement of the investigator)
- there are two or more clinically significant AEs considered to be at least possibly related to the IMP (based on the judgement of the investigator) and which are deemed to have impacted to a significant degree on the safety or physical or mental integrity of the affected subject(s), or which have resulted in permanent discontinuation of dosing in the affected subject(s)

If the study is halted, a substantial amendment must be submitted to the competent authorities. Likewise, approval from the competent authorities must be sought before the study can be resumed.

The clinical trial may be terminated at any time, if participation in the trial is no longer considered safe by the Principal Investigator (PI), the sponsor, the ethics committee (EC) or the MHRA.

8.7 Temporary contraindications

The below conditions are to be considered temporary contraindications to the trial products in the trial and are assessed on the day of each vaccination:

- Oral temperature > 38.3°C measured immediately before the vaccination
- Acute illness as judged by the investigator
- Additionally, with IN administration of trial vaccine: nasal congestion or nasal bleeding

If the subject is experiencing one of these conditions, the vaccination must be postponed until they have resolved. On the rescheduled visit the inclusion and exclusion criteria must be reassessed and the subject should be checked for the above temporary contraindications. If the subject is found to be healthy and eligible, the vaccine can be administered maintaining the planned intervals.

8.8 Investigational events

The trial consists of a total 10 regular visits to the clinical research site and five follow-up telephone calls to the subjects (**Table 8-2**).

8.8.1 Immunogenicity assessments

To measure anti-CTH522 IgG seroconversion, defined as a significant increase relative to baseline serum IgG level of the individual at any time point. Anti-CTH522 IgG will be determined using ELISA at SSI. The criteria for seroconversion are specified in the statistical analysis plan to be finalised prior to the unblinded review.

The immunogenicity will further be determined using a panel of exploratory assays performed at SSI and Imperial College as outlined in **Table 8-3**.

Table 8-3 Immunogenicity assessments

**Visit – immunology**		**1**	**2**	**3**	**4**	**5**	**6**	**7**	**8**	**9**	**10**
**Day**		**-90 to -1**	**0**	**14**	**28**	**42**	**112**	**126**	**140**	**154**	**168**
**Visit window**			**0**	**±2**	**+3**	**±2**	**+7**	**±2**	**+2**	**±2**	**±7**
**Assay used in secondary trial objective and performed at SSI:**											
Anti-CTH522 IgG ELISA (1, 4)	Serum tube 1x10 ml		X		X		X	X	X	X	X
**Exploratory assays performed at SSI:**											
Anti-central epitope IgG ELISA											
Anti-CTH522 IgA ELISA											
Anti-CTH522 IgG subtypes	Serum tube 1x10 ml		X		X		X	X	X	X	X
Neutralising antibodies against serovars D-G											
Anti-CTH522 IgG avidity (3)											
**Exploratory assays performed at Imperial College London**											
Vaginal anti-CTH522 IgA ELISA	Soft cup		X		X		X	X	X	X	X
Vaginal anti-CTH522 IgG ELISA	Soft cup		X		X		X	X	X	X	X
Nasal anti-CTH522 IgA ELISA	Nasal strip		X		X		X	X	X	X	X
Nasal anti-CTH522 IgG ELISA	Nasal strip		X		X		X	X	X	X	X

**Visit – immunology**		**1**	**2**	**3**	**4**	**5**	**6**	**7**	**8**	**9**	**10**
**Day**		**-90 to -1**	**0**	**14**	**28**	**42**	**112**	**126**	**140**	**154**	**168**
**Visit window**			**0**	**±2**	**+3**	**±2**	**+7**	**±2**	**+2**	**±2**	**±7**
Ocular anti-CTH522 IgA ELISA	Ocular strip		X		X		X	X	X	X	X
Ocular anti-CTH522 IgG ELISA	Ocular strip		X		X		X	X	X	X	X
Memory B cell Elispot (Frozen PBMCs)	Heparin tube 3 x 6 ml		X		X		X	X	X	X	X
ICS Flow (2) (Frozen PBMCs)	Heparin tube 3 x 6 ml		X	X	X	X	X	X	X	X	X
B cell cloning assay (Frozen PBMCs)	Heparin tube 8 x 6 ml								X		
**Total blood drawn for immunogenicity**	**ml**	**0**	**56**	**18**	**56**	**18**	**56**	**56**	**104**	**56**	**56**
**Total blood drawn for safety (5)**	**ml**	**13**	**8**	**8**	**8**	**8**	**8**	**8**	**8**	**8**	**8**

NOTES
(1): Secondary trial objective.
(2): ICS flow panel to be determined. Peptides provided by SSI
(3): Standard operating procedure (SOP) provided by Imperial College London
(4): To establish an in-house reference serum pool, 1 ml serum for each subject from day 126 sampling must be shipped to SSI in batches of 10, as soon as 10 subjects have completed visit 7 at day 126. These samples will be anonymized before transport
(5): 10% extra blood will be collected to account for variation

8.8.2 Safety assessments

The subjects will remain at the clinical site for 60 minutes after each vaccination so that any immediate AEs observed may be recorded. It is also documented if NO immediate AEs occurred. Following this observational period, the subjects may leave the clinical site. Site staff will ensure that the subjects have a diary card, ruler to measure and thermometer to enable them to record adverse events while away from the clinical site.

The subjects will be asked to fill in the diary card, every day, for 14 days after each vaccination, all of the solicited AEs experienced (if any).

From 14 days after each vaccination until the next vaccination, the subjects will be asked to fill in details in the diary, ONLY, if they experience an AE or if they take medication prescribed by a physician.

All information from the diary will be assessed and transferred to the eCRF by relevant trial staff.

8.8.3 Collection and handling of samples

Detailed information about collection and handling of blood, ocular, nasal and vaginal samples will be provided to investigator site staff in a Laboratory Manual.

In the course of this trial, an approximate volume of 85 ml blood will be collected for laboratory screening and safety tests, and 476 ml blood will be collected for immunogenicity assessment (please see **Table 8-3** for further information about immunogenicity and sampling).

Samples for screening and safety tests will be analysed at the investigator site (Imperial College Healthcare NHS Trust).

Ocular, nasal and vaginal secretion samples will be stored at the investigator site before being shipped in batches to Imperial College London for analysis.

Blood for PBMC-based analyses will be shipped unprocessed, as soon as possible after collection, to Imperial College London.

All samples to be analysed at SSI will be processed to serum at the investigator site and stored there until being shipped to SSI at the end of the trial and no later than 2 weeks after the last subject's last visit (LSLV). The samples must be shipped on dry ice with storage conditions monitored during the transport to SSI by use of a temperature logger. Documentation of the dispatch of samples from the investigational site and the receipt of the samples at SSI should be in accordance with the relevant SSI procedures for dispatch and receipt of samples.

It is the responsibility of the investigative site to pack the samples according to the above.

It is the responsibility of trial site to arrange the transport from the trial site to SSI.

8.8.3.1 Research biobank

At the research biobank at SSI the serum samples will, after the finalisation of the clinical trial be stored for a maximum of 15 years. The samples may be used for the purposes of standardisation, quality control and for future assays related to Chlamydia vaccine research and development. The samples will only be identifiable by subject number. The subject will be informed of this procedure in the information sheet and consent will be sought.

8.9 Laboratory assays

A pregnancy test will be performed by analysis of a urine sample for Human Chorionic Gonadotrophin (HCG) collected from female subjects at screening, the day of each vaccination and at the final safety visit (Visit 10, day 168). The analysis will be conducted by a member of the trial team at the clinical site – i.e. Imperial Clinical Research Facility.

Peripheral blood and urine will be collected and analysed at the laboratory at the clinical site for the following parameters at the time points specified in **Table 8-2**, and at additional time points if indicated to further evaluate or follow up AEs:

Blood:

- Renal (UE) Profile (sodium, potassium, chloride, urea, creatinine)
- Liver Function Tests (ALT, alkaline phosphatase, total bilirubin, total protein, albumin)
- Full Blood Count (WBC, RBC, haemoglobin, haematocrit, lymphocytes, monocytes, eosinophils, neutrophils, basophils and platelets

FDA guidelines (Toxicity Grading Scale for healthy Adult and Adolescent Volunteers Enrolled in Preventive Vaccine Clinical Trials Sep 2007) (Appendix 1) – adapted to local normal reference ranges – will be used by the investigator for grading the intensity of AEs including lab-related AEs.

8.10 Time schedule and recruitment

Submission of clinical trial application (CTA) to MHRA:	Q1 2016
Submission of CTA to EC:	Q1 2016
First subject's first visit (FSFV):	Q2 2016
Last subject's last visit (LSLV)*	Q1 2017
Analysis of serum samples completed:	Q2 2017
Final integrated clinical trial report (CTR):	Q3 2017

(*) The end of the trial is defined as LSLV, and the EC and MHRA will be notified within 90 days of the date of LSLV.

9 Investigational medicinal products

The investigational medicinal products (IMP) will be manufactured, filled and labelled according to good manufacturing practices (GMP) at SSI.

Saline will be used as placebo and will be normal saline for injection (ward stock, sourced commercially, individual single use vials).

9.1 Treatments administered

Three different trial vaccines and placebo will be administered in the 3 groups of subjects (**Table 8-1**):

- CTH522 chlamydia antigen adjuvanted with CAF01 for IM administration (preferably the non-dominant arm)
- CTH522 chlamydia antigen adjuvanted with aluminium hydroxide, $Al(OH)_3$, for IM administration (preferably the non-dominant arm)
- CTH522 chlamydia antigen diluted with Tris buffer for IN administration
- Placebo for IM and IN administration – i.e. saline.

9.2 Doses and administration

9.2.1 Doses

[redacted] The lowest amount of CTH522 antigen needed to elicit a protective immune response in humans is unknown.

A literature review of recently published vaccine trials, involving IM immunisation of recombinant proteins, shows that well tolerated doses range from 5-160 µg (14-19), and generally increasing the dose from 5-30 µg to 80-100 µg will increase the antibody titres (18, 19). However, a further increase in dose does not seem to have an effect on the antibody titre (15, 18).

SSI therefore chose to evaluate a CTH522 antigen dose of 85 µg for IM immunisation in the first in human trial. For IM immunisations SSI has adopted a classical prime-boost vaccination regime for the generation of antibodies in humans, consisting of a priming (two vaccinations 4 weeks apart), followed by a boosting vaccination after 12 weeks. This protocol is designed to generate high titred primary antibody response following priming and allow enough time for the antibody affinity maturation process before boosting after 12 weeks (20).

Pre-clinical data supports a vaccination protocol employing systemic immunisation followed by IN immunisations with unadjuvanted antigen to generate mucosal IgA, both in mice and pigs. Individual antigen dose for IN immunisation resulting in mucosal IgA in pigs was 50 µg (section 6.1.9 in the IB) and 60 µg (SSI study number 14R-SSI-001). Previous clinical experience with IN immunisations using 50-100ug of unadjuvanted antigen have shown it to be safe (TMUVA-01 trial - EudraCT Number: 2005-005140-81). Therefore, SSI selected a dose of 60 µg for IN immunisation.

Therefore, 85 µg CTH522 antigen and adjuvant ($Al(OH)_3$ and CAF01) were tested in a non-clinical Good Laboratory Practice toxicity study including 50 minipigs with 3 x IM and 2 x IN administrations. No treatment-related adverse clinical signs were recorded and no evidence of systemic toxicity was found. Compared to CTH522 alone CTH522 with either $Al(OH)_3$ or CAF01 induces significant higher level of anti-CTH522 IgG antibodies measured by ELISA.

CAF01: The CAF01 dose was based on the results from the ACAF01-01 trial – a CAF01 dose-escalation trial completed in the Netherlands. The results of the ACAF01-01 trial have been reported and published and conclude that a 625 µg/125 µg CAF01 is the optimal dose (12).

$Al(OH)_3$: The $Al(OH)_3$ dose was based on standard use in other vaccines – including the Malaria trial (PACTR registration number: ATMR2010060002033537)

9.2.2 Administration

- Each IM administration in the CAF01 group will consist of 85 µg CTH522 with 625 µg/125 µg CAF01 in a total volume of 0.6 ml.
- Each IM administration in the $Al(OH)_3$ group will consist of 85 µg CTH522 with 0.42 mg $Al(OH)_3$ in a total volume of 0.6 ml.
- IN administrations in both the CAF01 group and the $Al(OH)_3$ group will consist of two successive administrations (one in each nostril) of 30 µg CTH522 without adjuvant in a volume of 0.25 ml. Thus, a total CTH522 dose of 60 µg will be administered intranasally.
- Final formulation of trial vaccine is performed at the clinical site following the Vaccine Management Manual (Appendix 2) prepared by SSI
- IN administration will be performed using VaxINator™ delivered by SSI
- Subjects in each group will be vaccinated only if there are no safety concerns raised 24 hours after the first subject in each group receives the first vaccination. For the first block (block size = 7) only one volunteer will be vaccinated per day

Each vial and cardboard box of IMP is labelled with the content of the vial as referred to in the Vaccine Management Manual (Appendix 2). The vaccines may only be administered by authorised staff as indicated in the clinical site signature and delegation form. The identity of the injected trial

vaccine to a subject will thus be known by the clinical site staff dispensing and/or injecting the trial vaccine to the subject and by the trial monitor

However, the identity of the trial vaccine administered will remain unknown to the subject, the staff performing the safety assessments at the clinical site, the staff performing the immunology analysis at SSI and the staff at [redacted] performing the statistical safety and immunogenicity analysis until the time of database lock for the end-of-trial statistical analysis, respectively.

9.3 Composition of trial vaccines when reconstituted

The trial vaccines are composed as described below.

CTH522-CAF01 (IM)

Ingredient	Per dose volume 0.6 ml		Function
**CTH522**	85	µg	Active substance
[redacted]	[redacted]	[redacted]	[redacted]
[redacted]	[redacted]	[redacted]	[redacted]
**DDA**	[redacted]	[redacted]	Adjuvant
**TDB**	[redacted]	[redacted]	Adjuvant
**Water for injection**	up to 0.6	ml	Diluent

* The clinical doses are 625 µg and 125 µg for DDA and TDB respectively, but due to on-site preparation the actual doses are as written in the table.

CTH522-Al(OH)$_3$ (IM)

Ingredient	Per dose volume 0.6 ml		Function
**CTH522**	85	µg	Active substance
[redacted]	[redacted]	[redacted]	[redacted]
[redacted]	[redacted]	[redacted]	[redacted]
**Al(OH)$_3$**	[redacted]	[redacted]	Adjuvant
**Water for injection**	up to 0.6	ml	Diluent

**The clinical dose for Al(OH)$_3$ is 0.43 mg, but due to on-site preparation the actual dose is as written in the table

CTH522 (IN)

Ingredient	Per dose volume 0.25 ml*		Function
**CTH522**	30	µg	Active substance
[redacted]	[redacted]	[redacted]	[redacted]
[redacted]	[redacted]	[redacted]	[redacted]
**Water for injection**	up to 0.25	ml	Diluent

*0.25 ml to be injected per nostril

Placebo (IM)
0.9% saline (NaCl) stock purchased commercially.

9.4 Packaging and labelling

The IMP are all filled into vials (inner packaging) and are packed in cardboard boxes (outer packaging) at SSI according to GMP.

The labels for inner- and outer packaging will be produced at SSI according to the *'EU Guidelines to Good Manufacturing Practice Medicinal Products for Human and Veterinary Use Annex 13, Investigational Medicinal Products.* Labels will be printed at SSI.

9.5 Storage information

The IMP must be stored according to the protocol in a locked place at the clinical site under the responsibility of the site PI.

The expiry dates of the IMP will be indicated on the labels of the cardboard boxes. The IMP should not be used after the indicated dates.

The Tris diluent, CAF01 adjuvant and $Al(OH)_3$ adjuvant must be stored in a refrigerator at + 2°C to + 8°C. The CTH522 antigen must be stored at -15 to -25°C.

It is considered critical that theCAF01 adjuvant and $Al(OH)_3$ adjuvant are NOT exposed to freezing.

The refrigerators must be equipped with thermometers and the temperature must be controlled on a daily basis and documented in the storage condition logs.

The PI/pharmacist is responsible for the IMP. Relevant clinical site staff will monitor the storage conditions of the IMP during the trial.

In case of deviations in storage conditions as judged by either the site responsible investigator or the clinical trial monitor, the clinical trial manager at SSI must be contacted to decide if the IMP can be used in the trial or must be replaced.

9.6 Transport of IMP

The IMP will be released by a qualified person (QP), and subsequently transported to the clinical site when the clinical trial has been approved by the competent authority (CA) and by the relevant ethics committee (EC).

The transport of the IMP will be arranged according to standard operating procedures at SSI.

Temperature loggers will be used for monitoring the storage conditions during the transport.

Procedures will be in place for documenting the dispatch of IMP from SSI and the receipt of the IMP at the clinical site.

During the clinical trial, the monitor will check that the dispatch/receipt documentation in the investigator’s file is adequate and correct.

The transfer of IMP to the clinical trial site is covered in Material Transfer Agreement (Appendix 3)

9.7 Randomisation procedure

The randomisation will be performed via the eCRF system.

Randomisation data is kept strictly confidential, accessible only to authorised persons, until the time of un-blinding.

Only when the trial has been completed and the data closed, the codes will be broken and made available for data analysis.

9.8 Blinding and unblinding procedure

Blinding will be obtained by shielding the subjects from seeing preparation of the trial drug and by having unblinded trial personnel not involved in any trial assessments (efficacy or safety) responsible for preparing and administering the trial drug. This unblinded member of staff will prepare, and administer trial drug. Further this unblinded member of staff will be the only one doing trial drug accountability.

During trial drug administration a blinded member of staff also shielded from seeing trial drug or any procedures related hereto will be present. This blinded member of staff will observe the subject and monitor any adverse events during or after trial drug administration.

All used material will be removed by the unblinded member of staff without revealing the infused fluid.

Trial drug accountability will be monitored by an unblinded clinical research associate (CRA).

Breaking the Blind Prior to Trial Completion

The PI and delegates at the participating centre will have access via the system 'eClinicalOS' to unblind individual subjects, in order to obtain information to trial drug administered, in case of a medical emergency.

The investigator should only unblind the treatment allocation to a subject during the clinical trial if this is relevant to the safety of the subject. The identity of the IMP would be revealed by the PI or designee, for that subject only.

It may be necessary for [redacted] to unblind a subject's treatment for the purpose of expedited reporting to the Competent Authorities and/or Research Ethic Committee. In these situations [redacted] will keep the unblinding from PI and trial site staff, subject as well as SSI personnel involved in monitoring, data analysis and interpretation.

Unblinding of a subject is performed in the 'eClinicalOS' system under the' information tab' and is only possible for persons who have obtained rights by SSI to perform unblinding. Only authorised persons will have access to this part of the system. When unblinding is performed, the reason for the unblinding is requested. The date of unblinding, reason and the person performing the unblinding is saved via audit-trail.

A screen-dump of the 'information tab' page including the information of the unblinded subject, reason for unblinding, date and person performing the unblinding, is made and printed. This print is filed in an envelope with the subject's allocation number and sealed with a label. The date and name of the person performing the unblinding is added the label and the envelope is kept in a secure designated place.

At the end of the trial, the code envelopes will be returned to the sponsor.

In case of a break-down of the 'eClinicalOS' system, a designated person at [redacted], who is not otherwise involved in any study related work, can be contacted to obtain information of the trial drug allocated to any specific subject.

If a subject's investigational product code is broken, the subject will be permanently withdrawn from investigational product, but will be followed as indicated in Section 8.6.

9.9 Treatment compliance

As this clinical trial is investigating vaccines that are administered by the staff at the clinical site, the issue of subject treatment compliance will be addressed by evaluating the vaccine administration data recorded by the staff in trial-related documents/logs.

During the clinical trial, the monitor will check that vaccine administration data is recorded correctly.

9.10 Drug accountability

It is the site responsibility that all unused and used vaccines are accounted for during the trial and documented in the relevant trial documents/logs, and that unused trial vaccines are returned to SSI after completion of the trial.

During the clinical trial the monitor will check that drug accountability is maintained. Empty vials and vaccine cardboard boxes are kept at the clinical site until the termination monitoring visit. At the end of the trial monitor will ensure that all unused vaccines have been returned to SSI by signing the accountability log.

9.11 Precautions and overdosing

Anaphylactic reactions are a potential risk; therefore adrenaline and a resuscitation kit and instructions for use must be kept available for immediate use in the room where the injections are taking place. Medical staff trained in resuscitation must be in the clinic for the vaccination and 60 minutes thereafter.

Vasovagal syncope (i.e. fainting), is sometimes seen in relation to vaccination and blood sampling. It will be attempted to minimise the occurrence of this reaction, by allowing the Subject to lie down during administration of vaccine and blood draw.

After both IM and IN administration of the vaccine, the subject must stay at the trial site for 60 minutes under observation. Before the subject leaves, immediate adverse events (if any) are recorded in the eCRF by delegated staff. It is also recorded if NO immediate AEs occurred.

9.12 Concomitant medication

Vaccination with a vaccine received within 14 days of the start of the trial or before any on the immunisation visits in the trial is not allowed.

Immunosuppressive agents - e.g. inhaled, nasal or injected corticosteroid - may not be administered during the trial. Topical steroid are allowed, unless applied to the IM injection site.

Antipyretics/analgesics may not be taken within 24 hours prior to the day of vaccination. If given the vaccination should be postponed. Please see section 8.5.

Furthermore, during the first 72 hours after the vaccination, antipyretics/analgesics should preferably be avoided. If taken, the details should be recorded in the diary card and in the concomitant medication pages of the eCRF.

Other medications (except for vitamins and minerals) are recorded in diary cards by the subjects as instructed and subsequently transferred to the eCRF by relevant trial staff. Please see section 8.5.

9.13 Drug interactions

Specific drug interactions are not known or expected.

10 Ethical aspects

The background of the present clinical trial is to investigate the safety and immunogenicity of the chlamydia vaccine.

SSI has taken the initiative to conduct the present GCP clinical trial and is the GCP sponsor of the trial. The trial is partly funded by a grant from ADITECH. The grant covers expenses to materials, staff at SSI and at the investigator's site and the CROs managing data management, statistics and pharmacovigilance on behalf of SSI. Work items outsourced from SSI to investigator's site and the CROs are covered by signed financial agreements and Task Orders.

The trial will be conducted in accordance with the latest version of Declaration of Helsinki (21) and ICH-GCP

An application to conduct the trial will be submitted to MHRA and the Ethics Committee for approval.

The subjects will receive verbal and written information about the purpose of trial and its nature, including details of any potential risks.

The total amount of blood required from each volunteer throughout the trial period of 6 months will be approximately 561 ml as compared to 450 ml normally drawn from blood donors. Blood samples will be coded before the blood samples are sent for laboratory analyses. Only the investigator and his medical collaborators will have access to information that may link laboratory results with personal identification.

The formulation and filling of the drug product is performed in a classified fill and finish facility by the Process and Technology Implementation unit at SSI. The manufacturing, formulation, filling and packaging procedures are performed according to GMP at SSI

There was no evidence of toxic effect of CTH522-CAF01 and CTH522-$Al(OH)_3$ when administered to minipigs in a more intensive regimen than anticipated in the clinical program. All non-clinical information supports the suitability of CTH522-CAF01 and CTH522-$Al(OH)_3$ as a safe, immunogenic, and possibly effective vaccine to induce immunity in *C. trachomatis* uninfected individuals.

The safety and reactogenicity of the IMP is expected to be comparable to the safety observed in previous clinical trials conducted where the CAF01 and Alum adjuvants have been tested with other vaccine antigens in Phase I clinical trials.

10.1 Risks and inconveniences

The following adverse events may occur:

Between 1 and 10 % of the Subjects are expected to experience injection site reactions, such as pain, tenderness, redness and swelling and itching and/or systemic reactions, such as skin rashes, general malaise and/or fever (> 38.3°C). Rare reactions include lymphadenopathy, high fever (≥ 40ºC), hypersensitivity, urticaria and nodules or sterile abscesses at the injection site. Uncommon reactions include nausea and diarrhoea.

Anaphylactic reactions are very rare. However, appropriate medical treatment and trained staff will be available in case an anaphylactic reaction occurs.

Vasovagal syncope (i.e. fainting), may occur in this population of subjects. It will be attempted to minimise the occurrence of this reaction, by allowing the subjects to lie down during administration of vaccine and blood drawings.

The trial personnel performing the procedures will inform the subjects on the procedures prior to blood drawings and injection.

10.2 Benefits

This is a healthy volunteer trial, so there will be no direct health benefit to individual subjects. However, they will undergo a general health and STI screen. STIs may be asymptomatic, and if diagnosed the subjects will be counselled and offered referral to the confidential service provided by their local NHS GUM clinic.

If trial results are satisfactory, further clinical trials will be initiated and may lead to a marketed vaccine against *C. trachomatis.*

The Subjects will receive payment to compensate them for their time, inconvenience, travel expenses etc. They will be paid £100 per scheduled clinic visit, to a maximum of £1000, paid as a lump sum at the end of participation. Volunteers who attend screening but who are not enrolled will not receive payment.

10.3 Overall conclusion risks and benefits:

Based on general considerations, experience from clinical trials with CAF01 and marketing experiences with vaccines adjuvanted with $Al(OH)_3$, the IMPs to be used in the present trial, is expected to be safe.

11 Adverse events

11.1 Definition and terms

The following definitions are in accordance with the EU Guidance (CT-3) (2011/C 172/01) and European Clinical Trial Directive 2001/20/EC of 4 April 2001 (22)

Adverse Event (AE)
An adverse event (AE) is any untoward medical occurrence in a subject administered a medical product and which does not necessarily have to have a causal relationship with the administered product.

An (AE) can therefore be any unfavourable and unintended sign (including an abnormal laboratory finding, for example), symptom or disease temporally associated with the use of a medical product, whether or not considered related to the medicinal product.

The definition covers also medication errors and uses outside what is foreseen in the protocol, including misuse and abuse of the product.

Adverse Reaction (AR)

An adverse reaction (AR) is any untoward and unintended response to an investigational medicinal product related to any dose administered.

Unexpected Adverse Reaction

An AR, the nature or severity of which is not consistent with the investigator's brochure (IB).

Unsolicited AEs:

These will be sought from each vaccination, through non-leading AE questioning such as 'how have you been feeling', and through symptom-directed physical examination. Unscheduled laboratory testing and other investigations may be performed as required to investigate AE

Serious Adverse Event (SAE) or Serious Adverse Reaction (SAR)

An SAE or SAR is any untoward medical occurrence that at any dose:

- results in death
- is life-threatening*
- requires hospitalisation or prolongation of existing hospitalisation
- results in persistent or significant disability/incapacity
- is a congenital anomaly or birth defect
- is an important medical event (IME)**

NOTES:
*The term 'life-threatening' refers to an event where the subject was at risk of death at the time of the event, it does not refer to an event which hypothetically could have caused death, had it been more severe.

**The term 'IME' refers to a medically important event that does not meet any of the SAE criteria, but may require medical or surgical consultation or intervention to prevent one of the other serious outcomes listed in the definitions above.

Suspected Unexpected Serious Adverse Reaction (SUSAR):

A SUSAR is a suspected unexpected serious adverse reaction according to the definitions given above.

11.2 Collection and Recording of AEs and SAEs

11.2.1 Collection

The source of AEs covers for example the subject's response to questions about her health (a standard non-leading question), information from diary card, symptoms spontaneously reported by a subject, investigations and examinations where the findings are assessed by investigator to be clinically significant changes or abnormalities, other information relating to the subject's health becoming known to the investigator (for example hospitalisation).

From each vaccination until the visit scheduled 14 days later, specific AEs will be solicited through the use of diary cards and predefined laboratory testing:

- Injection site reactions to IM administration: erythema, pain, tenderness, warmth, pruritis, stiffness and swelling
- Local reactions to IN administration: discharge, congestion, discomfort, sneezing, cough.
- Systemic reactions to IM/IN vaccines: abnormally raised temperature, chills, myalgia, malaise, fatigue, rash, headache, nausea and vomiting; and clinically significant abnormal values among full blood count, liver function test and renal profile results

11.2.2 Recording

The investigator must record all AEs, non-serious and serious, expected and unexpected in the relevant form of the eCRF. This starts from the signed informed consent for participation in the trial until the end of trial visits.

The date of onset is the date when the first sign(s) or symptoms where first noted. If the AE is an abnormal clinically significant laboratory test or outcome of an examination the onset date is the date the sample was taken or the examination was performed.

If an AE or SAE is still ongoing or the outcome is unknown at the end of the clinical trial at her last visit, it must be followed up by the investigator until it has been resolved or stabilised.

All SAEs must be recorded on the Serious Adverse Event form, please see Appendix 4. If the investigator has unblinded the trial drug allocation of the subject, the screen-dump from the unblinding is forwarded together with the completed Serious Adverse Event (SAE) form.

If an SAE is entered in the eCRF/eClinicalOS system, the system automatically forwards an e-mail to [redacted] and SSI [redacted] as a pre-notification.

11.2.3 Assessments

The investigator must assess the characteristics of the event(s) according to below criteria:

Seriousness criteria

For the assessment of the seriousness of the adverse event, please see definition under section 11.1 'SAE or Serious Adverse Reaction'.

Causality assessment

The causal relationship between an AE and the trial product must be assessed by the investigator using the following terms:

Not related: An AE that is definitely NOT related to the product administered

Possible: An AE with a reasonable time relationship to product administration, but which can also be explained by concurrent disease, other drugs or other cause.

Probable: An AE with a reasonable time relationship to product administration, and which is unlikely to be attributed to concurrent disease, other drugs or other cause.

Certain: An AE occurring in a plausible time relationship to product administration and which cannot be explained by concurrent disease, other drugs or other cause.

A Suspected Adverse Reaction is defined as an AE which has been assessed as possibly, probably or certainly related to the product administered (i.e. which has a reasonable suspected causal relationship to the product administered).

Expectedness:

Expectedness is assessed according to the Reference Safety Information section in the newest version of the Investigator's Brochure. Since there is no clinical experience available for CTH522, CTH522-CAF01 or CTH522-Al(OH)$_3$, no expected adverse events are listed in the Reference Safety Information. Hence all related SAEs in this trial should be reported as SUSARs.

Intensity:

The intensity of an AE must be assessed by the investigator using the FDA guidance (23). Where a specific AE is not listed in the guidance, its intensity must be assessed by the investigator using the following terms:

- **Mild (Grade 1):** No interference with daily activity
- **Moderate (Grade 2):** Some interference with daily activity not requiring medical intervention.
- **Severe (Grade 3):** Prevents daily activity and requires medical intervention.
- **Potentially life-threatening (Grade 4) :** Emergency visit or hospitalisation

Outcome:

The outcome of an AE must be assessed by the investigator using the following terms:

- Recovered/resolved
- Recovered/resolved with sequelae
- Recovering/resolving
- Not recovered/not resolved
- Fatal
- Unknown

11.3 Expedited reporting of adverse event

If a SAE occurs, immediate reporting is required by completing the Serious Adverse Event form, please see Appendix 4.

The site PI must submit the completed SAE form, as soon as possible, and latest within 24 hours of first knowledge, to both JPM and SSI.

The SAE form should be sent password protected by e-mail to:

- [redacted]

  e-mail: [redacted]

  [redacted]

- SSI

  e-mail:[redacted]

  [redacted]

Any changes to the listed contact information after the final version of the protocol will be documented separately from the protocol and circulated accordingly.

All related SAEs must be reported as SUSARs on an expedited basis by [redacted] to the MHRA and the Research Ethic Committee according to the below listed timelines:

- For fatal or life-threatening SUSAR reports, notification as soon as possible, but no later than seven calendar days [7] after first knowledge of the event and final reporting after an additional eight [8] calendar days.
- For other SUSARs reporting, as soon as possible, but no later than fifteen [15] calendar days after first knowledge of the event.

The blinded SUSAR reports will be archived in the trial master file at SSI and in the investigator´s file.

All Adverse Events and Adverse Reactions will be submitted to the CA in the final clinical trial report (CTR).

A DSUR should be prepared in accordance with ICH Guideline E2F on Development Safety Update Report at yearly intervals and submitted to the MHRA.

11.4 Data safety monitoring board

A Data Safety Monitoring Board (DSMB) will be established for the trial and will advise SSI on safety issues in accordance with the DSMB charter. The DSMB comprised by individual experts will advise the investigator and sponsor.

The membership of the DSMB will reflect the disciplines and medical specialties necessary to interpret the data from the clinical trial and to fully evaluate participant safety. No member of the DSMB should have direct involvement in the conduct of the clinical trial. Furthermore, no member should have financial, proprietary, professional, or other interests that may affect impartial, independent decision-making by the DSMB.

The members of the DSMB serve in an individual capacity and provide their expertise and recommendations in accordance to a DSMB charter. The primary responsibilities of the DSMB are to 1) periodically review and evaluate the accumulated trial data for participant safety, trial conduct and progress and 2) make recommendations concerning the continuation, modification, or

termination of the trial. The DSMB considers trial-specific data as well as relevant background knowledge about the disease, test agent, or patient population under trial.

12 Trial Steering Committee

A Trial Steering Committee will be established consisting of [redacted] from UK and from SSI [redacted]. The Trial Steering Committee will be responsible for the day-to-day running and management of the trial and will have regular TCs and face-to-face meetings.

13 Data management and statistical analysis

13.1 General considerations

Data management and statistical analysis and reporting will be performed by a CRO on behalf of SSI. The CRO will be specialised in data management and statistics.

13.2 Data management

The trial is run as a Electronic Data Capture (EDC) trial, i.e. relevant data is entered by the sites directly into the clinical database. Electronical transferred data will be handled by [redacted]. The database and application are set up and managed by the [redacted]. The eCRF is designed to capture all required information in compliance with GCPs standards. The eCRF system is hosted by [redacted] a full service, global eClinical and EDC software and services company, Safe Harbour certified.

All data, except laboratory data, will be collected using an eCRF compliant with 21 CFR Part 11 regulation. Laboratory data will be collected via the local laboratories and transferred to the eCRF by the trial staff at the site. Data management will be performed in accordance with applicable standards and data cleaning procedures. Only authorised access to the eCRF will be possible using encrypted username and password. Roles in the system are given according to functions. All tasks performed in the eCRF are logged in an audit trail.

The eCRF will contain validation checks according to the Trial Validation Plan (TVP) to maintain an ongoing quality check of data entered. All data validation will be performed as part of the system.

The Investigator will approve the data using an electronic signature and thereby confirm the accuracy of the data recorded.

Medical History and AEs will be coded using the MedDRA dictionary. Concomitant medication will be coded using the WHO Drug dictionary. Further details about trial setup and closure will be documented in the Data Handling Protocol (DHP).

13.3 Clean file procedures

Before releasing the database for statistical analyses and clinical trial reporting, the data manager will ensure that all quality control procedures in connection with data capture, cleaning and reconciliation of data have been finalised and documented.

The data manager is responsible for setting up a clean file meeting with relevant trial team members in accordance with [redacted] SOP.

13.4 Coding of AEs and concomitant medication:

AE shall be coded according to the current MedDRA version with respect to System Organ Class, High Level Group Term, High Level Term and Preferred Term.

Concomitant medication shall be coded according to the WHO DRUG ATC coding system.

Coding of AE and Concomitant medication shall be entered into the trial database.

13.5 Analysis populations

The basis for safety analysis is the Safety analysis set. The safety analysis set consist of all subjects randomised and exposed to study drug, including subjects who are withdrawn after exposure to trial drug.

The analysis of primary endpoints and all safety endpoints will be based on safety analysis data set.

Full Analysis Set (FAS) which include all observed data for all randomised subjects.

A per-protocol analysis set will be defined as subjects completing the trial without deviations judged to influence the primary endpoints.

Additional analyses of primary endpoints will be performed using the PP analysis set, unless the discrepancy between Safety data and PP analysis set is less than 10%.

The analysis sets will be defined at the data base lock (DBL) meeting.

13.6 Statistical methods

Baseline is defined as the last assessment with available data prior to the first administration of trial medication.

Categorical data will be summarised descriptively by treatment, using number and percentages of subjects. Continuous data will be presented using the number of subjects (n), mean, standard deviation (SD), median, lower quartile, upper quartile, minimum and maximum. Both the absolute values and the change from baseline will be presented.

Descriptive statistics for all endpoints will be presented by treatment group and visit (if applicable).

All statistical tests will be carried out as two-sided and performed on a 5 % significance level unless otherwise stated. Estimated treatment differences and 95 % CIs will be presented together with the corresponding p-value.

13.6.1 Demographics and other baseline characteristics

The three treatment groups will be compared descriptively with respect to disposition, demography, baseline characteristics (age and race), medical history and concomitant medication, using both the

safety analysis set and the FAS. However, if the difference between Safety analysis Set and FAS is two subjects or less, then only the Safety analysis set will be used

13.6.2 Safety analysis

The binary primary endpoints are

- Solicited injection site reactions after IM (any of the following: erythema, pain, tenderness, pruritus, warmth, stiffness and swelling)
- Solicited local reactions after IN administration (discharge, including bleeding, congestion, discomfort, sneezing and cough)
- Solicited systemic reactions (abnormally raised temperature, chills, myalgia, malaise, fatigue, rash, headache, nausea and vomiting, and clinically significant abnormal values among full blood count, liver function test and renal profile results)

The frequencies of the binary endpoints will be presented with corresponding 95% CI.

These endpoints will be analysed by pairwise comparison of active treatment groups versus Placebo in 2 by 2 tables (presenting number and percentage of subjects experiencing the criteria) using Fishers exact test. Also the two active treatment groups will be compared similar.

Adverse Drug Reaction will be presented descriptively by treatment group (number of subjects, percentage of subjects experiencing the event and number of events), in tables of SOC and preferred Terms in summary tables presenting each group in separate columns.

Vital signs are presented descriptively by treatment group and visit. Both absolute values and changes from baseline are presented both in tables and in Box-plots by visit.

13.6.3 Immunogenicity analysis

Percentage of subjects achieving seroconversion for anti-CTH522 IgG antibody at any time points after vaccination(s) will be descriptively presented. The criteria for seroconversion will be specified in the statistical analysis plan by [redacted] and SSI. The statistical analysis plan will be finalised prior to the unblinded review.

13.7 Sample size determinations

Estimation within treatment group of event probabilities:

Assume we estimate the 95% CI for event probability in an active treatment group, where 12 of 15 subjects experienced an event of the type considered. Considering (example) 0.8 as a proportion of

events observed in an active group then the estimated frequency with 95% CI would be 80% [52%; 96%] which is considered a relevant size of CI for the purpose of this trial.

N	Proportion	Confidence interval	
15	0.47	0.21	0.73
15	0.6	0.32	0.84
15	0.8	0.52	0.96

13.8 Interim analysis

Interim analysis will be done as per the DSMB charter.

13.9 Handling of missing data

No impute of missing data will be applied. Observed data will be used for analysis.

13.10 Multiplicity

The placebo group is used for comparison with each of the active treatment groups and the two active treatment groups will also be compared. No adjustment of the level of significance is planned, because the correlations are not known, but the nominal p-values should be considered with caution and seen as screening measures of magnitude of differences.

The trial is exploratory in its nature and therefore no adjustment of level of significance is planned.

14 Good clinical practice considerations

14.1 ICH GCP / Declaration of Helsinki

The clinical trial will be performed in accordance with the latest version of the Declaration of Helsinki (24)

14.2 Subject information and informed consent

14.2.1 General procedures for obtaining informed consent

The subjects will be recruited through the clinical site's healthy volunteer database, posters and information meetings at universities and other public forums, advertisements in newspapers and social media. All written recruiting material will be approved in advance by the concerned ethics committee.

Prior to the first trial-related visit, the potential subject are given ample time to reflect on the trial information. At the first visit, the subject information and consent form (Appendix 5) are reviewed thoroughly with the subject. The verbal review is always conducted by a physician or nurse, and encompasses:

- A comprehensible presentation of background and objectives of the clinical trial
- A presentation of the trial design (randomised)
- Any predictable benefits, risks, adverse reactions, inconveniences, complications and disadvantages
- Potential risks
- The right to reflection
- The right to waive information on clinical trial results
- Description of the Investigational Medicinal Products
- That collection and extradition of information to/from the subject's subject files in the Health Care System may occur
- Remuneration and reimbursement of transportation expenses
- Questions from the subject

The subjects will always be given the opportunity to discuss participation with a physician prior to first dosing.

It is emphasised to the subject, that participation is voluntary and that the subject at any time can withdraw from the trial. The potential subject is informed that data collected during participation in the trial is confidential.

The review of the written information with the subject and the signing of the consent form take place under undisturbed environment. Should there be a need for further reflection before signing the consent form, the potential subject is offered another 24 hours to reflect. A new appointment is made for the signing of the consent form.

Once the consent form is signed, medical examination and other trial related activities can be performed.

14.3 Ethics committee submission and approval

The PI and the sponsor will collaborate on the submission of the clinical trial application to the EC.

An electronic initial submission form is completed on www.myresearchproject.org.uk. The PI and the sponsor must sign the submission form and send to the EC as a pdf file together with the other required documentation.

14.4 Competent authority submission and approval

A request for a clinical trial authorisation will be submitted for approval to the MHRA in the UK

The application will be submitted by SSI as GCP sponsor of the trial.

Screening or inclusion of Subjects will only be initiated after approval by both the EC and CA, and also by the clinical site's local NHS R&D Office.

14.5 Subject data protection

The PI at clinical site is responsible for keeping a log of all Subjects. All subjects who are subject to at least one trial intervention must be listed in the Subject Screening Log. For screening failures the reason for exclusion must be stated in the Subject Screening Log.

The log will identify the subjects by full name, full address, date of birth, screening number, randomisation number (for included subjects) and subject initials and must be kept in the Investigator File.

In the eCRF, only the screening number, the randomisation number and birth date will be recorded. It is, however, possible to trace back any eCRFs to the subject logs, in case needed.

At completion of the trial, normally at the site closure monitoring visit, the site PI must date and sign off the subject log(s) for completeness.

14.6 Investigator’s responsibilities

The site PI is responsible for:

- The conduct of the clinical trial in accordance with the protocol, current GCP guidelines, and other applicable national requirements and regulations.
- Documenting the receipt, storage, dispensing and accountability of IMP as appropriate.
- All data including AEs being recorded in the eCRFs and for the immediate reporting of serious AEs to SSI as described in section 11
- Being available to answer or clarify any protocol related queries from the CA or ECs
- Keeping a log of all subjects
- The PI undertakes this responsibility by signing the Investigator Statement Form (Appendix 6)

If the site PI delegates his/her responsibilities to other staff members, it has to be documented in the clinical site signature and delegation form. This documentation has to be completed before trial initiation, which is to be updated during the trial and to be signed off for correctness at the end of the trial.

14.7 Curricula vitae

Before trial initiation, SSI must collect current, dated and signed curriculum vitae for all participating investigators and other relevant trial staff including monitors and data managers.

14.8 Indemnity statement

Prior to initiation of the clinical trial, SSI will present an indemnity statement to the investigator(s) signed by the director of Vaccine Development. Please see Appendix 7.

14.9 Training

The site PI is responsible for adequate training of the investigators and other trial staff at his/her site in the protocol, other trial relevant documents and ICH GCP, before working with trial related activities. SSI will provide the PI and other trial personnel with relevant instructions and information needed for the conduct of this clinical trial in accordance with the current GCP guidelines and this Protocol. The information and training will be given prior to the start of the trial.

A certificate for each attendee will be issued and kept in the Investigators' Files and copies in the trial master file at SSI.

14.10 Monitoring

Monitoring will be performed by SSI according to SSI GCP SOPs and a trial specific monitoring plan.

The monitor will check:

- That the protocol is being followed
- That facilities and staffing remain acceptable
- That the eCRFs are being correctly filled in
- That the eCRFs are in accordance with source data
- That the clinical supplies can be accounted for
- That the serum samples, and the vaccines are stored properly
- That the Investigators' File is being kept in proper order
- All visits must be documented.
- Any query must be discussed and resolved with the PI

A pre-trial initiation visit will be performed at the site, followed by a trial initiation visit. Subsequent routine monitoring visits will be performed during the trial with approximately 4-6 weeks interval. At trial completion, a close-out monitoring visit will be performed

14.11 Audit and inspection

The clinical site must give access to Quality Assurance staff from SSI for the conduct of audits and to inspectors from the MHRA or other relevant competent authority for the conduct of inspections. Auditors and inspectors must have access to all trial-related documents, including the subject identification log(s) and/or personal medical records.

14.12 Definition of source data

Source data is defined as all information (e.g. clinical findings, observations, other activities in a clinical trial) in original records and certified copies of original records necessary for the reconstruction and evaluation of the clinical trial. Source data are contained in source documents.

At the clinical site, a document, which identifies all (expected) source documents, will be prepared (and signed off by the site PI) before the initiation of the trial.

Examples of clinical site source documents are:

- EC and CA approval documents
- Signed informed consent forms
- The subject screening log / subject identification code list
- Investigator's or nurse's notes/worksheets (for data not recorded or not recorded directly in the eCRF)
- The eCRF for data recorded directly in the eCRF
- Completed diaries
- Completed CIOMS reporting forms of serious AEs/reactions
- Vaccine inventory and accountability log(s)
- Serum sample inventory and accountability log(s)
- Logs of monitoring of storage conditions of vaccines and serum samples

It is the responsibility of the site PI to archive all source data/documents for at least 15 years after the termination of the clinical trial. Entries in the eCRF will be verified with source documentation. The review of medical records will be performed in a manner to ensure that subject confidentiality is maintained.

The trial monitor from SSI will check the eCRF for accuracy and completion and perform source data verification (SDV). The trial monitor will document SDV of all reviewed sections of the eCRF

14.13 Archiving of essential documents

It is the responsibility of SSI to maintain all GCP essential documents, as defined in ICH GCP for at least 15 years after the date of the last subject's last visit in the trial.

The following GCP essential documents must always be archived under the responsibility of the PI:

- Informed Consent, Confidentiality of Data, and Acceptance of Terms of Insurance Forms of all subjects
- Potential Participation log

- Screening log
- Completed eCRFs
- Diaries
- Completed reporting forms of Serious Adverse Events
- Certified copies of source documentation

The completeness of the investigator's file with the GCP essential documents will be documented as part of the site-closure monitoring visit.

15 Agreement and financial settlement

An agreement between SSI and the clinical site will be signed off prior to inclusion of the first subject in the trial. These agreements will clearly state the rights and obligations of the concerned parties and are the legally binding documents between the parties. This protocol, in its current version, will be an appendix to the agreement with the clinical site. The agreement must be signed by the legal representatives of the parties prior to trial start.

Imperial Hospital must grant the PI permission to conduct the trial on its premises. This permit must be signed prior to the inclusion of the first subject in the trial.

16 Budget

This trial is funded by a grant from the European Community's European 7th Framework Program, ADITEC (HEALTH-F4-2011-18 280873). The results of the trial will be published according to the dissemination strategy as detailed in the ADITEC consortium agreement.

17 Insurance

SSI is the sponsor and manufacturer of the trial products to be administered in this clinical trial. SSI carries a product liability insurance programme including cover for clinical trials. The insurance programme covers worldwide and is currently placed with insurer [redacted]. The policy covers claims arising from injury/injuries caused by trial medication used in this clinical trial sponsored by SSI, if the trial product has been used in accordance with the instructions given in the protocol. The insurance certificate is enclosed in Appendix 8.

18 Confidentiality and disclosure

All eCRF data, information and results generated by SSI, as well as information on product development, patented or not, including patent applications and manufacturing processes not previously published, are considered confidential and shall remain the sole property of the SSI.

A CTR, will be prepared by SSI and will be reviewed and approved by the trial statistician and the PI.

No data from the clinical trial, unless approved by SSI in writing, may be published, presented or communicated, except to MHRA or EC, prior to the issue of the final CTR unless approved by SSI in writing. The names of authors and their order of appearance in the publication is stated in the agreement between SSI and the PI.

19 Protocol amendments

The trial procedures may be changed if the PI and the sponsor agree to the changes. If the changes are substantial, MHRA and EC must be notified and approve the changes prior to implementation in accordance with local regulations. All substantial changes must be documented by protocol modifications and rewritten full protocols, if applicable.

20 References

1. ECDC. Chlamydia. Factsheet for health professionals. 2015.
2. Brunham RC, Rappuoli R. Chlamydia trachomatis control requires a vaccine. Vaccine. 2013;31(15):1892-7.
3. Farley TA, Cohen DA, Elkins W. Asymptomatic sexually transmitted diseases: the case for screening. Preventive medicine. 2003;36(4):502-9.
4. Li W, Murthy AK, Guentzel MN, Chambers JP, Forsthuber TG, Seshu J, et al. Immunization with a combination of integral chlamydial antigens and a defined secreted protein induces robust immunity against genital chlamydial challenge. Infection and immunity. 2010;78(9):3942-9.
5. Olsen A, Follmann F, Erneholm K, Rosenkrands I, Andersen P. Vaccine promoted neutralizing antibodies directed to the VD4 of MOMP protect against Chlamydia trachomatis infection and upper genital tract pathology. The Journal of infectious diseases. 2015.
6. Brunham RC, Kuo CC, Cles L, Holmes KK. Correlation of host immune response with quantitative recovery of Chlamydia trachomatis from the human endocervix. Infection and immunity. 1983;39(3):1491-4.
7. Nunes A, Nogueira PJ, Borrego MJ, Gomes JP. Adaptive evolution of the Chlamydia trachomatis dominant antigen reveals distinct evolutionary scenarios for B- and T-cell epitopes: worldwide survey. PloS one. 2010;5(10).
8. Igietseme JU, Eko FO, Black CM. Chlamydia vaccines: recent developments and the role of adjuvants in future formulations. Expert review of vaccines. 2011;10(11):1585-96.
9. Garcon N, Chomez P, Van Mechelen M. GlaxoSmithKline Adjuvant Systems in vaccines: concepts, achievements and perspectives. Expert review of vaccines. 2007;6(5):723-39.
10. Lindenstrom T, Woodworth J, Dietrich J, Aagaard C, Andersen P, Agger EM. Vaccine-induced th17 cells are maintained long-term postvaccination as a distinct and phenotypically stable memory subset. Infection and immunity. 2012;80(10):3533-44.
11. Frenzel A, Hust M, Schirrmann T. Expression of recombinant antibodies. Frontiers in immunology. 2013;4:217.
12. van Dissel JT, Joosten SA, Hoff ST, Soonawala D, Prins C, Hokey DA, et al. A novel liposomal adjuvant system, CAF01, promotes long-lived Mycobacterium tuberculosis-specific T-cell responses in human. Vaccine. 2014;32(52):7098-107.
13. Lycke N. Recent progress in mucosal vaccine development: potential and limitations. Nature reviews Immunology. 2012;12(8):592-605.
14. Sagara I, Ellis RD, Dicko A, Niambele MB, Kamate B, Guindo O, et al. A randomized and controlled Phase 1 study of the safety and immunogenicity of the AMA1-C1/Alhydrogel + CPG 7909 vaccine for Plasmodium falciparum malaria in semi-immune Malian adults. Vaccine. 2009;27(52):7292-8.
15. Ellis RD, Wu Y, Martin LB, Shaffer D, Miura K, Aebig J, et al. Phase 1 study in malaria naive adults of BSAM2/Alhydrogel(R)+CPG 7909, a blood stage vaccine against P. falciparum malaria. PloS one. 2012;7(10):e46094.
16. Otsyula N, Angov E, Bergmann-Leitner E, Koech M, Khan F, Bennett J, et al. Results from tandem Phase 1 studies evaluating the safety, reactogenicity and immunogenicity of the vaccine candidate antigen Plasmodium falciparum FVO merozoite surface protein-1 (MSP1(42)) administered intramuscularly with adjuvant system AS01. Malaria journal. 2013;12:29.

17. Wressnigg N, Barrett PN, Pollabauer EM, O'Rourke M, Portsmouth D, Schwendinger MG, et al. A Novel multivalent OspA vaccine against Lyme borreliosis is safe and immunogenic in an adult population previously infected with Borrelia burgdorferi sensu lato. Clinical and vaccine immunology : CVI. 2014;21(11):1490-9.
18. El Sahly HM, Patel SM, Atmar RL, Lanford TA, Dube T, Thompson D, et al. Safety and immunogenicity of a recombinant nonglycosylated erythrocyte binding antigen 175 Region II malaria vaccine in healthy adults living in an area where malaria is not endemic. Clinical and vaccine immunology : CVI. 2010;17(10):1552-9.
19. Jepsen MP, Jogdand PS, Singh SK, Esen M, Christiansen M, Issifou S, et al. The malaria vaccine candidate GMZ2 elicits functional antibodies in individuals from malaria endemic and non-endemic areas. The Journal of infectious diseases. 2013;208(3):479-88.
20. Siegrist C-A. Vaccine immunology. In: Stanley Plotkin WOaPO, editor. Vaccines. 62013. p. 14.
21. Helsinki WMADO. Ethical Principles for Medical Research Involving Human Subjects 2013.
22. EMA. Detailed guidance on the collection, verification and presentation of adverse event/reaction report arising from clinical trials on medicinal products for human use. CT-3/2011/C 172/01. 2011.
23. FDA. Guidance for Industry. Toxicity grading scale for healthy adult and adolescent volunteers enrolled in preventive vaccine clinical trials. FDA, CBER September 2007. 2007.
24. ICH. ICH Topic E2A. Guidance on clinical safety data management: Definitions and standards for expedited reporting (CPMP/ICH/377/95). 1994). 1994.

21 Appendices

Appendix 1: FDA guidelines

Appendix 2: Vaccine Management Manual

Appendix 3: Material Transfer Agreement

Appendix 4: SAE form

Appendix 5: Informed consent form

Appendix 6: Investigator Statement Form

Appendix 7: Indemnity Statement

Appendix 8: Insurance certificate
